# Exploring the Darkverse: A Multi-Perspective Analysis of the Negative Societal Impacts of the Metaverse

**DOI:** 10.1007/s10796-023-10400-x

**Published:** 2023-06-02

**Authors:** Yogesh K. Dwivedi, Nir Kshetri, Laurie Hughes, Nripendra P. Rana, Abdullah M. Baabdullah, Arpan Kumar Kar, Alex Koohang, Samuel Ribeiro-Navarrete, Nina Belei, Janarthanan Balakrishnan, Sriparna Basu, Abhishek Behl, Gareth H. Davies, Vincent Dutot, Rohita Dwivedi, Leighton Evans, Reto Felix, Richard Foster-Fletcher, Mihalis Giannakis, Ashish Gupta, Chris Hinsch, Animesh Jain, Nina Jane Patel, Timothy Jung, Satinder Juneja, Qeis Kamran, Sanjar Mohamed AB, Neeraj Pandey, Savvas Papagiannidis, Ramakrishnan Raman, Philipp A. Rauschnabel, Preeti Tak, Alexandra Taylor, M. Claudia tom Dieck, Giampaolo Viglia, Yichuan Wang, Meiyi Yan

**Affiliations:** 1 Digital Futures for Sustainable Business & Society Research Group, School of Management, Swansea University, Bay Campus, Fabian Bay, Swansea, Wales UK; 2 Department of Management, Symbiosis Institute of Business Management, Pune & Symbiosis International (Deemed University), Pune, Maharashtra India; 3 Bryan School of Business and Economics, University of North Carolina at Greensboro, Greensboro, NC USA; 4 Department of Management and Marketing, College of Business and Economics, Qatar University, Doha, Qatar; 5 Department of Management Information Systems, Faculty of Economics and Administration, King Abdulaziz University, Jeddah, Saudi Arabia; 6 School of Artificial Intelligence, Indian Institute of Technology Delhi, Hauz Khas, New Delhi, India; 7 Department of Management Studies, Indian Institute of Technology Delhi, Hauz Khas, New Delhi, India; 8 School of Computing, Middle Georgia State University, Macon, GA USA; 9 ESIC University, Madrid, Spain; 10 University of Economics and Human Sciences, Warsaw, Poland; 11 Institute for Management Research, Radboud University Nijmegen, Nijmegen, The Netherlands; 12 Department of Management Studies, National Institute of Technology, Tiruchirappalli, India; 13 FORE School of Management, New Delhi, India; 14 Management Development Institute, Gurgaon, India; 15 School of Management, Swansea University, Swansea, UK; 16 EM Normandie Business School, Métis Lab, 30-32 Rue Henri Barbusse, 92110 Clichy, France; 17 Prin. L. N. Welingkar Insititute of Management Development and Research, Mumbai, India; 18 Department of Media and Communication, Swansea University, Swansea, UK; 19 Robert C. Vackar College of Business & Entrepreneurship, University of Texas Rio Grande Valley, 1201 W University Dr, Edinburg, TX 78539 USA; 20 Morality and Knowledge in Artificial Intelligence (MKAI), Milton Keynes, UK; 21 Audencia Nantes Business School, 8 Route de La Jonelière, B.P. 31222, 44312 Nantes, Cedex 3 France; 22 Marketing Area, Indian Institute of Foreign Trade (IIFT), New Delhi, India; 23 Seidman College of Business, Grand Valley State University, 1 Campus Dr, Allendale, USA; 24 Government Relations & Policy at MKAI, New Delhi, India; 25 Kabuni and University of Reading, Reading, UK; 26 Faculty of Business and Law, Manchester Metropolitan University, Manchester, UK; 27 School of Management, Kyung Hee University, Seoul, South Korea; 28 Birlasoft Limited, Marketing Area, Indian Institute of Foreign Trade (IIFT), New Delhi, India; 29 Department of International Management, Dortmund, Germany; 30 Department of Engineering Technology, University of Twente, Enschede, Netherlands; 31 Eikonikos, Dubai, United Arab Emirates; 32 Marketing Area, National Institute of Industrial Engineering, Mumbai, India; 33 Newcastle University Business School, Newcastle Upon Tyne, UK; 34 Symbiosis Institute of Business Management, Pune & Symbiosis International (Deemed University), Pune, India; 35 Digital Marketing and Media Innovation, College of Business, Universität Der Bundeswehr München, Werner-Heisenberg-Weg 39, 85577 Neubiberg, Germany; 36 Faculty of Business and Law, Manchester Metropolitan University, Manchester, UK; 37 School of Strategy, Marketing and Innovation, University of Portsmouth, Portland Street, Portsmouth, PO13DE UK; 38 Department of Economics and Political Science, University of Aosta Valley, Aosta, Italy; 39 Sheffield University Management School, The University of Sheffield, Sheffield, UK; 40 Film Producer of Jindian Warner Pictures Beijing Co. LTD, Beijing, China

**Keywords:** Dark side of the metaverse, Metaverse, Negative consequences, Second life, Unintended consequences, Virtual reality, Virtual world

## Abstract

The Metaverse has the potential to form the next pervasive computing archetype that can transform many aspects of work and life at a societal level. Despite the many forecasted benefits from the metaverse, its negative outcomes have remained relatively unexplored with the majority of views grounded on logical thoughts derived from prior data points linked with similar technologies, somewhat lacking academic and expert perspective. This study responds to the dark side perspectives through informed and multifaceted narratives provided by invited leading academics and experts from diverse disciplinary backgrounds. The metaverse dark side perspectives covered include: technological and consumer vulnerability, privacy, and diminished reality, human–computer interface, identity theft, invasive advertising, misinformation, propaganda, phishing, financial crimes, terrorist activities, abuse, pornography, social inclusion, mental health, sexual harassment and metaverse-triggered unintended consequences. The paper concludes with a synthesis of common themes, formulating propositions, and presenting implications for practice and policy.

## Introduction

A decade ago, the metaverse was within the realms of science fiction, and has become a reality via the advancement of technology (Dwivedi et al., 2022a). The metaverse has been synonymously used to depict the future state of the internet and a new and immersive user experience and engagement (Dwivedi et al., 2023a). The metaverse is a simulated environment that is developed to converge an enhanced version of physical and virtual realities (Ball, 2022; Dwivedi et al., 2022a, a). The adoption of the metaverse could be significant across various verticals such as gaming, social platforms, and commerce (Wiederhold, 2022a, b). Amidst various benefits and experiences expected from the metaverse, its negative consequences remain relatively unknown (Dwivedi et al., 2022a).

The metaverse is built by integrating various technologies such as virtual reality, augmented reality, 3D modelling, animation, cloud computing, blockchain, artificial intelligence (AI), and next-generation internet applications (Dwivedi et al., 2022a, a). While previous research has asserted that these technologies may impact human life in many ways, there exists a lack of debate on the negative aspects of these technologies within the context of an immersive metaverse. Given that these technologies are now integrated with a single domain, what are the negative consequences for users and the wider population? This question is yet to be explored comprehensively. Unlike most technological advancements, the metaverse has the potential to augment and even replace the physical interaction with an immersive virtual experience. There is the possibility that the metaverse can broadly experience a range of threats in the areas of privacy, ethical, legal, social, and personal security. The literature on the metaverse is increasing, however, many studies seem somewhat preoccupied with a positive metaverse narrative, perhaps ignoring the negative impacts of widespread adoption (Belk et al., 2022), highlighting a need to provide a holistic understanding of the dark sides that can exist in the metaverse.

The metaverse is created and operated via the integration of several technologies categorised at peripheral and functional levels and previous research has identified the limitations of these technologies at an individual level. Tian et al. (2018) explained that prolonged usage of extended reality (XR) or wearable devices would affect the user's health. Other studies have highlighted that AI-based simulated environments can gradually affect the cognitive processing skills of the users (Dwivedi et al., 2021b). Dwivedi et al. (2023b) highlight that data ownership in the blockchain can be a threat depending upon the network. Hashizume et al. (2013) described the security issues that can be present in the cloud computing system. Weingartner (2021) explains the social inequalities that may be present in the next generation of the internet. Dwivedi et al. (2021a) highlight the lack of privacy and social security on social networking sites. The metaverse can emerge as a consolidated platform by combining these technologies within a defined ecosystem. But can developers and brands that create the metaverse to address each technology's threat, or will it be compromised for the experience and benefits it can provide?

Although academic researchers have yet to fully explore the dark side of the metaverse, recognised sources and technology experts have started providing their perspectives (Analyticsinsight.net, 2022; Forbes, 2022; PewResearchCenter, 2022). Most views are grounded with logical thoughts deriving from historical data points associated with similar technologies. The discussion on the dark side of the metaverse can be seen from two viewpoints: technology and user perspectives. From the technology side, the technical design and functionalities can be the source of many user-related issues and user behaviour may vary, resulting in negative consequences within the metaverse. Although there is evidence of the circumstances in which metaverse gamers have encountered abuse and racism (IndiaTimes, 2022), most experts predict that the metaverse can lead to a different world with no doctrines or legal commitment entitled to the users (PewResearchCenter, 2022). Apart from the consequences of technology impacting the metaverse, users can create a separate identity with minimal legal or ethical consequences. These behaviours can subsequently build an unsafe environment for other users. Despite the hype and positive narrative on the growth potential of the metaverse, it is critical that decision makers do not ignore the detrimental consequences to users and wider society. Some vulnerable users may find it hard to differentiate between reality and the virtual world where the trauma and abuse suffered in metaverse worlds could cause real physical harm and negative consequences. There are many questions that need answers which will allow future research on the metaverse to orient their perspectives to benefit all stakeholders of the metaverse.

This study addresses the gaps in research on the metaverse dark side from a holistic perspective by inviting and collating the contributions from a number of experts in this area. The implications of this paper will benefit academia and practice by framing appropriate measures to first identify and then offer solutions to the many negative aspects of the metaverse. This study addresses the numerous challenges and difficulties in the metaverse, including mental health and well-being, ethical and moral issues, privacy and security, and diminished reality. This research work follows the approach of von Foerster (2003) to consolidate the perspectives and structure them to arrive at a holistic meaning. The implications of this research have yielded an understanding of the explored and unexplored areas in the dark side of the metaverse, which has allowed us to list future research avenues in this area.

The remaining sections are presented in the following sequence: Section 2 includes the contributions provided by the experts. The contributions individually are brief but exhaustive in explanation. Based on the understanding derived from Section 2, Section 3 discusses and highlights prospective future directions. Consolidating from contributions, Section 4 provides the concluding remarks.

## Multiple Perspectives from Leading Marketing Experts

In line with an approach originally proposed by von Foerster (2003) and subsequent multiple perspectives-based studies (Dwivedi et al., 2015, 2020, 2021a, b, 2022a, b, c, 2023a, c), we explore a number of diverse perspectives on the dark side of the metaverse. The individual contributions provided by the experts, is based on their own perspectives on the negative aspects of the metaverse and unintended consequences from its deployment, adoption, and widespread use. Table 1 presents the full list of experts and title of their contributors.

**Table 1:** Individual contributions

Contribution Title	Author(s)
**Contribution 2.1:** Darkverse: Exploring the challenges and difficulties in metaverse	Janarthanan Balakrishnan, Rohita Dwivedi and Sriparna Basu
**Contribution 2.2:** Metaverse- a space of known-knowns and known-unknowns	Qeis Kamran
**Contribution 2.3:** Is it time to be all in on the Metaverse?	Giampaolo Viglia
**Contribution 2.4:** Critical Perspectives on the Metaverse: True Value, Privacy, and Diminished Reality	Philipp Rauschnabel, Reto Felix and Chris Hinsch
**Contribution 2.5:** Human – Computer Interface in Metaverse: Perspectives from Dark Side	Satinder Juneja, Preeti Tak & Ashish Gupta
**Contribution 2.6:** A study of the potential dark sides of the metaverse in order to make immersive experiences more secure	Sanjar Mohamed AB
**Contribution 2.7:** The Impact of Metaverses on Social Inclusion and Wellbeing	Savvas Papagiannidis
**Contribution 2.8:** Darkside of Metaverse: Mental Health and Wellbeing Perspective	Alexandra Taylor, M. Claudia tom Dieck, and Timothy Jung
**Contribution 2.9:** Sexual Harassment in the metaverse: Will Extended Reality exacerbate toxicity online?	Animesh Jain & Richard Foster-Fletcher
**Contribution 2.10:** Mitigation Strategies of Metaverse-trigged Unintended Consequences	Yichuan Wang & Meiyi Yan
**Contribution 2.11:** Scoping the future of Dark Side of Metaverse – A Thematic Overview	Abhishek Behl, Gareth H. Davies and Vincent Dutot
**Contribution 2.12:** Murky side of metaverse: Implications and research questions	Neeraj Pandey, Mihalis Giannakis & Ramakrishnan Raman
**Contribution 2.13:** The darkside of the metaverse today	Leighton Evans
**Contribution 2.14:** Children, Young People and the Metaverse	Nina Jane Patel

The 14 expert perspectives presented in the remaining part of this section are presented largely in unabridged form. The inherent unevenness with this approach is countered by capturing the unique perspectives from each of the experts as they focus on key aspects of dark side of the metaverse (Dwivedi et al., 2022a, b, c, 2023a, c).

### Darkverse: Exploring the challenges and difficulties in metaverse—Janarthanan Balakrishnan, Rohita Dwivedi and Sriparna Basu

The metaverse has the potential to bring dreams to reality. From volcanoes to the sea, the metaverse can bring anything to reality with the help of the right tools and technology (Dwivedi et al., 2023a). Despite the benefits and virtualism that the metaverse can offer, there exists another perhaps more negative side to the metaverse. Although multiple viewpoints have articulated a positive narrative on the metaverse, at best, most are still individual perspectives. As is the case with many other technological advancements, the metaverse does have many limitations. However, compared to other immersive and interactive technologies, the metaverse can have substantial adverse consequences if not appropriately managed and controlled via effective governance (Xi et al., 2022). The darkverse is a recent term used to explain the organised crime that can take place within the virtual space (Analyticsinsight.net, 2022). We explain and extend the darkverse as a terminology that denotes the disadvantages of the metaverse. Most of the news articles discuss darkverse as a separate platform which can be used for online crime, without realising that darkverse can be present within the ecosystem of the metaverse in many ways. Of the negative effects present in the metaverse, some can be minimised by rigorous and timely intervention or policy directions, whereas the other effects may become exponentially more prominent as the penetration of the metaverse increases. The remaining sections of 2.1 discusses the adverse effects of the metaverse.

**Fig. 1:**
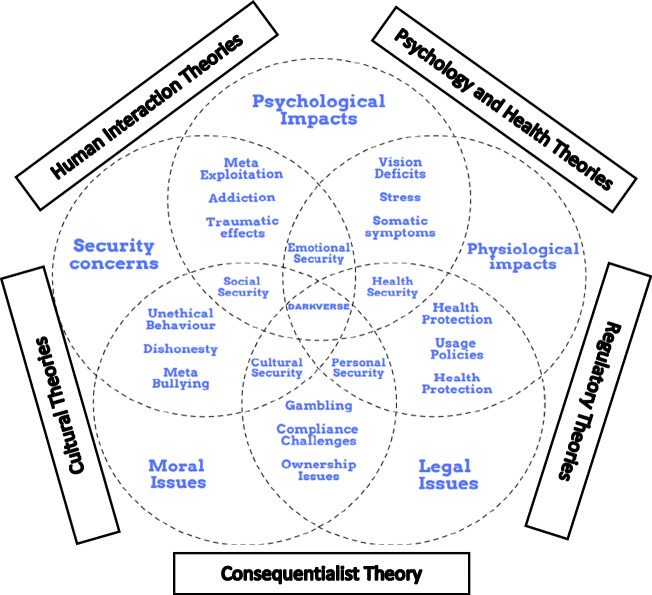
A holistic model of the Darkverse

Figure 1 explains the five major consequences (themes) of the metaverse namely; psychological impacts, physiological impacts, security concerns, moral issues, and legal issues. These five factors were derived from the academic and practice-based literature. The issues mentioned in the intersection of two themes denotes consequences that arise in connection with the metaverse. For example: The topics meta exploitation, addiction, and traumatic effects can be a part of both security concerns and psychological impacts in varying contexts. The figure also explains five integrated security issues namely; emotional security, health security, personal security, cultural security, and social security which are holistically derived based on the major themes and their intersecting consequences. For example: social security is an intersectional derivation from the angle of psychological impacts, security concerns, and moral issues. Thus, the circles overall show the major themes and their interactive elements that can consequentially affect the users of the metaverse. These consequences can be addressed and explored in future studies with the use of holistic or specific theoretical setup, as given in the outer layer of the figures. These theories can be used in research to investigate these variables proposed inside the figure in detail.

#### Psychological and Physiological Impacts

Human psychology is conditioned to the physical entity and its operational boundaries. During the last decade, extended reality devices have given humans a taste of a virtual entity. Research in cyberpsychology has explained that humans harvest pleasure and experience from virtual space at the cost of physical and mental health. In the metaverse, the benefits and flow of experience that users derive, can result in a psychological and physiological costs (Usmani et al., 2022a, b). XR devices have been proven to affect human health in areas such as vision and addiction (Rauschnabel et al., 2022b). Regular usage of the metaverse can create blurred consciousness between reality and virtuality (Dwivedi et al., 2023a) and research that has investigated virtual gaming has explained similar effects. In the long run, these effects can generate stress and build more psychological and physiological discomfort. Forbes (2022) states that the metaverse poses significant long term mental health risks for users. CNBC (2022b) highlights that the metaverse can be more addictive and, if unprotected, may create a traumatic experience for vulnerable users. The study asserts that children could get more addicted to the metaverse, highlighting whether the metaverse can promise health and emotional security for users. Although it is too soon to ask these questions, users adopting similar technologies using XR have suffered similar consequences. The success of the metaverse is hugely dependent upon its users' psychological and physiological wellbeing. Future research can employ psychology and health theories to investigate the consequences of the metaverse and subsequent impact on users.

#### Moral and Legal Issues

The regulatory mechanism of the metaverse is yet to be formulated or investigated (Kostenko et al., 2022). There is a necessity that the law in the metaverse needs well-defined regulations to enable legal and moral behaviour within the virtual space (BFSI Economic Times, 2022). There are incidents of cyberbullying, flirting, harassment, and racism in virtual games reported before; though the incidents seem more virtual, the trauma and effects are near to reality (Theguardian.com, 2022). Thus, there is an extended possibility that issues related to identity theft, gambling, and unethical behaviours can increase in the metaverse. Compared to psychological and physiological threats, legal threats are more likely to be within the control of the government and companies to create a more favourable ecosystem. Moreover, it is a question whether the metaverse can promise cultural and personal security. The moral and legal issues underlying the metaverse can potentially harm the global cultural and ethical ecosystem. Policy makers should be able to build a global legal ecosystem keeping the cultural, ethnic and user profiles in mind. Also, future research in the metaverse can employ consequentialist and regulatory theories to investigate these issues.

#### Security Issues

Since the inception of cyber transactions, security is always a more significant concern in virtual space (Gale et al., 2022). Security concerns are inevitably present in any advanced technology applications. Compared to other technologies, the metaverse offers more user behavioural tracking avenues to developers and marketers, such as: eye-tracking, pupil movements, geometric movements, physical movements, and so on (Techtarget, 2022). Security can be breached when the data is not used for the users' benefit but for the third party's benefit. Such a scenario is more likely in the case of the metaverse, where multiple tracking parameters and a free world environment can question the presence of privacy and data security. Moreover, the security issues can accelerate psychological and moral issues. For example: Given the question of personal security, meta bullying (PewResearchCenter, 2022) can be prominently present in the metaverse. Incidents of meta bullying can create traumatic experiences for users and also damage the moral significance within the metaverse ecosystem. An integrative operational ecosystem could benefit the metaverse. A centralised information flow using blockchains could be developed (Ali et al., 2023), enabling rigorous identity markers integrated with citizenship identities, and authorising digital currencies. The introductory stages of the metaverse are likely to be more challenging as governance controls are established and become stabilised over time. Users of the metaverse should be fully informed of the negative aspects within the virtual world. Future research can employ cultural and human-interaction theories to investigate user privacy expectations.

### Metaverse- a Space of Known-Knowns and Known-Unknowns—Qeis Kamran

The Metaverse as an emerging space of reality is still in the process of construction and evolution. While much research needs to be done to understand the risks and the dark side pertaining to the metaverse some observed dimensions are briefly treated here.

#### Collection and Processing of Biometric Data

The evolution of the metaverse has pushed the dimension of access to the users’ personal data to a new level. Capturing the biometric data in addition to eye tracking, capturing users’ emotional behavior and access to observation of the gait patterns of individuals, will enable contemporary tech giants as Meta (formerly known as Facebook), in addition to the other gaming platforms as Decentraland, Roblox, Sandbox, Fortnight etc., collecting all forms of data, thus enhancing their unpreceded power dynamics over their users. This information is gained in addition to the financial data and playing patterns of the individuals with access to larger network of contacts of the users and the themes of their discussions in their chatrooms. This large concentration of the users’ metadata with algorithmic machine learning powers would push the asymmetry of power to a much higher level of concentration as we have so far known, thus increasing the notion of surveillance capitalism beyond control and products of behavior futures designed to much enhance the addictive powers in terms of economics of attention and marketing of personal data to an ecosystem of data hungry tech firms.

#### Metaverse Service Providers are Completely Centralized

The terms of services rendered in the Metaverse are so far completely centralized and largely controlled by the firms operating the individual giant platforms. While, via the possibility of interoperability, control that could be reintroduced to the users in terms of choice of play, individual investments, doing business, ownership of land and digital assets and marketing, is still much in the far horizon. Hence each platform would first try to limit the notion of interoperability and thus increase switching costs so that more business is done within their own domains.

Furthermore, metaverse platforms have the power to expose their users to any kind of known and unknown challenges based on the possible risks of cyberattacks to their platforms, or to the partners in their greater ecosystem and value chains. The breach of such data could expose many users to a much higher level of risk such as fraud, cyber-hacking, identity theft and greater physical harm. Logistical and residential data could be used to cause harm and danger to the users and their families. Users may have much more difficulty in defending themselves in these situations. The dimensions of proving a breach of their data or cyber-bullying, cyber-hacking especially by foreign non-state actors (because of challenges in the design of a metaverse platform) is likely to be a very difficult undertaking. Significant expertise is likely to be needed in the case of a known breach of personal data to protect and compensate users. In addition to that the lack of legal protection and regulatory boundaries such as the General Data Protection Regulation (GDPR) framework for Web 3 spaces, will open the metaverse to all sorts of actors exploiting a dystopian creative destruction-based innovation modus operandi.

#### Dictating the Right to the Terms of Services

Metaverse platforms have the right to shape their terms and conditions of services in ways that will be more profitable to them, while locking their users in to their chosen boundaries. The regulatory governing authorities are still in a state of confusion over the metaverse. Hence the boundaries, wherein the whole emergence of the metaverse, assets owned in the metaverse as landownership, brand-equity, artistic and creative works etc., are not really defined. In a judgment from the European Court of Justice (ECJ) (Judgment of 22. 12. 2010 — Case C-393/09), it found that although these digital artefacts may not be considered as computer programs they can be still considered as works of art, hence be protectable by copyright regulatory frameworks, as long as these interfaces are the designers’ and authors’ own creative intellectual output. However, having to notify their users of any kind of transparent changes in their terms of conditions much digital assets could be frozen or just disappear. This could also result in a breach of wallet data of the users and before noticing much wealth and capital could change ownership to a non-tracible and non-detectable party in a country with very weak legal protection.

These mentioned cases create a high risk situations for the users where metaverse platforms are able to enforce full control over the users’ assets and overall financial security.

#### Ban and the Access to the Platforms

With much possibility of innovation and evolution in the Metaverse the notion of making use of the freedom of speech or of political opinion may be censured. This does not mean that illegal behavior of any kind should be tolerated under the umbrella of freedom of speech. But moreover, the platforms may sanction their users for opinions that they do not share and thus limit or deny access to their platforms. In addition, based on the many chatrooms and communicational possibilities much political influence could be taken place without any kind of regulation, transparency or control. The Facebook and Cambridge Analytica partnership has illustrated the possibility of data-sharing and micro-targeting of users not only without their consent but moreover by being subjugated to unlimited volume of psychologically driven political campaigns that are free of any kind of public scrutiny, even after the facts of abusive and illegal conduct are known. Thus, the damages will be irreversible, as is in the cases of Brexit, Bolsonaro’s conquest against the Amazonians or the Trump’s MAGA-cult danger to the US constitution and overall democracy in the world.

#### Artificial Scarcity

In essence, digital assets are not really scarce. The artificial scarcity by design may bring a good return on investments in the short term, but such dimensions and sources of revenue enhancement are still very novel and new. The artificial limitations of land or any kind of (digital) non-fungible token (NFT) provided as a twin by pairing it with a physical artifact, thus enhancing the artifact’s overall value or on its own without twinning, may flood the market and cause huge losses of value. There is still vital lack of understanding of what truly is the metaverse in terms of its evolution and who decides what limits are created. The limits the platforms have set are likely to support their own profit-oriented causes in a Friedmanian sense of the social responsibility of business to increase its profits (Friedman, 1970).

Furthermore, the experience of assets artificially created are yet very limited. To what extent are the changes of ownership of a platform as Elon Musk’s public attacks on the leaders of Twitter illustrated, will protect the users’ assets. What if by the change of ownership, the appetite for profits or tolerance towards risk, shift and change the conduct of the platform’s steering committee towards unknown or dangerous territories? These risks will be likely outsourced to users without them having any power over their resources, assets, brand equities and the conduct of the platforms.

#### Social Darwinism via Code

Algorithmic bias has been already recognized as a major flaw in the coding of artificial intelligence artifacts. “Algorithms themselves are a source of bias that is often overlooked in reviews of algorithmic bias, which tend to focus on discriminatory outcomes or data bias” (Kirkpatrick, 2016, p.16). Known cases of algorithmic bias (Stinson, 2022), could transfer real world problems into the metaverse. This could result in increasing social hierarchies and limiting access to diverse forms of capital (in a Bourdieuian sense). Algorithmic bias is based on an artificial distinction of social division of taste and habitus derived by a doxa in metaverse research and practice and new field of evolutionary social structuring derived from virtual spaces. The metaverse-based and artificially driven version of a utopian paradox breaks this doxa. By imagining a new design of a digital social world wherein: “anyone in whom there is a potential Raphael' of painting or politics could develop without hindrance, it forces one to see that the concentration of the embodied or objectified instruments of production is scarcely less in politics than in art, and prevents one forgetting all the potential Raphaels whom the mechanisms responsible for this monopoly are excluded much more effectively than any 'ideological state apparatuses” (Bourdieu, 1984, p. 398).

While much known risk dimensions are postulated, our cyber-physical global societies must be ready for the many unknown-unknowns that the emergence and evolution of metaverse may bring and cultivate those ambidextrous organizational capabilities to cope with them.

### Is it Time to be all in on the Metaverse?—Giampaolo Viglia

Some innovations take time to become profitable and mature. Others will never reach a mature stage. A clear example is the so-called Google Glass, a prototype of smart glasses that has failed to meet consumers’ expectations (Leonard, 2022).

Despite the amount of Google searches for the word “Metaverse” has exploded in recent times (EuPortal, 2022), the company Meta who went all in on the metaverse has lost a significant amount of capital, falling by more than 70% on the US stock market by October 2022. In what follows we discuss few technological issues and some consumer vulnerability issues that are slowing down the metaverse revolution.

#### Technological Issues

Although the metaverse has the potential to transform how consumers interact with brands, it is now only seen as an alternate reality that follows the same fundamental laws as the physical world (Dwivedi et al., 2022a). In this new reality we still face important hardware limits.

Three types of technology are necessary for the metaverse to function properly (Chen, 2022). First, complete digital duplication must be accomplished either by scanning or digital construction. Next, networks must create real-time renderings of those physical surroundings in the cloud or on devices to make them accessible. Display technologies that deliver a genuine real-world experience will be the third and most difficult.

The structures and tools that support this new era are already in place and will soon enable the metaverse to thrive. Virtual content, cellular networks, display technology, rendering engines, and processing power are all expanding to new heights. For instance, scanning technology costs a small fraction of what they did a few years ago.

The equipment needed to create a 3D model at the time cost hundreds of thousands of dollars. Structured light, for instance, "reads" a projected light pattern on an object, which is then recreated by scanning cameras that identify distortions brought on by differences in distance. 3D imaging makes use of a variety of supporting technologies. Another method, known as "Time of Flight," uses light pulses to illuminate a scene, which are then reflected and picked up by a sensor to identify and digitally incorporate moving objects.

#### Consumer Vulnerability Issues

A virtual and augmented reality lifestyle might cause loneliness. According to psychological studies, people desire to appear better than they actually are in order to enhance and evaluate their own selves (Strube & Roemmele, 1985). This is especially clear on social media, where users frequently compare themselves to others. We might begin to favour using virtual environments because they offer this idealized world. However, if we begin to choose a virtual existence, our real, imperfect lives may seem terrible, which would be detrimental to our confidence and self-esteem. Policymakers are left with unanswered questions about how to curb the spread of harmful interactions and fraudulent information. With this criticism, other stakeholders may definitely assist. These worries would be allayed, in particular, by a deliberate spoke design in collaboration with tech businesses.

The internet environment can also offer support to someone who has a condition or is trying to lose weight. Recent studies have already confirmed this conclusion (Fletcher-Brown et al., 2021). However, the metaverse can be harmful if it takes the place of physical activity and in-person social interactions that are good for mental health, such as developing physical relationships and getting enough sleep.

#### Conclusion

The new marketing platform of the future, known as metaverse, will be used to present and breathe life into a variety of companies in the 3D interactive digital environment. The metaverse is a virtual representation of how things are done in the real world. Users can interact with one another in this 3D virtual environment by using avatars that look like them. This will have a significant impact on how businesses use the marketing function going forward as well as how we interact with one another. While the metaverse can bring enterpreneurial opportunities, it can also affect the wellbeing of consumers, especially the most vulnerable ones. Additionally, there are few technological issues, such as hardware and software issues. This suggests that a full adoption of the metaverse will take some time to materialise.

### Critical Perspectives on the Metaverse: True Value, Privacy, and Diminished Reality – Philipp Rauschnabel, Reto Felix and Chris Hinsch

Most of the journal space devoted to the metaverse concept, especially within marketing, focuses on potential benefits for consumers and brands (e.g., Dwivedi et al., 2023a; Rauschnabel et al., 2022a). However, there are emerging critical voices that question these benefits and call for caution when interacting in this space. Unfortunately, the term metaverse is currently not consistently defined in the literature. We conceptualize the term metaverse as a fully immersive three-dimensional environment able to integrate physical and/or virtual worlds. As such, metaverse can be accessed through XR interfaces such as VR and AR (e.g., Dwivedi et al., 2023a; Rauschnabel et al., 2022b).

Metaverse platforms also tend to imitate communities or societies; for instance, many offer some sort of ownership (e.g., virtual space, non-fungible tokens), the presence of others (e.g., in form of avatars), activities (e.g., concerts, games etc.), payment methods (e.g., crypto currencies), laws (i.e., specific rules), freedom of movement (discussed as interoperability), certain structures (often some sort of decentralization, e.g., through blockchain technology), and a three-dimensional environment (typically in augmented and virtual reality – summarized as XR; Rauschnabel et al., 2022b). We note that strictly speaking, the “true” metaverse does not exist today (Dwivedi et al., 2023a). Emerging common platforms, such as VRChat, Mozilla Hubs, The Sandbox, Decentraland, Spatial.io, SuperWorld, and SecondLife are considered to be “metaverse-like”, and therefore, are often called “proto metaverses”. For simplicity, we use the term metaverse throughout this section.

The following paragraphs discuss three major potential challenges of the metaverse concept that are particularly relevant from a consumer and societal perspective: The value of virtual products, privacy risks, and challenges associated with diminished reality. We note that this list based on the very early stages of metaverse development, and our negative tone should not be taken as call for political actions to hinder metaverse developments. Rather, we argue that an early understanding of these negative aspects can complement prior research and lead to a more theoretically holistic and societally beneficial understanding of this domain. Furthermore, addressing potential drivers of consumer backlash is likely also in the interest of brands seeking to develop the metaverse (e.g., hardware firms, platforms, content creators, marketers, etc.), since such threats might limit consumer adoption or trigger political intervention.

#### A Critical Look on the Value of Virtual Content

Interoperable avatars, i.e., a single avatar that can represent a specific person across various applications (Dwivedi et al., 2022a; Park et al., 2022), is a concept that is central to a true metaverse environment. Participants will be much more likely to invest in aesthetically augmenting their virtual appearance if their avatar is persistent and can be used in multiple virtual or augmented spaces. This will likely stimulate the demand for branded virtual merchandise (e.g., branded virtual handbags, jewelry, or shoes). NFTs provide a technological means to clarify ownership of these virtual items (Hofstetter et al., 2022). NFTs should be distinguished from digital rights management or copyright protection as they signify true ownership stored through blockchain technology. Brands using NFTs can allow users to verify the originality of their products and prove that they are not counterfeits or unlicensed copies.

While these developments may open entirely new opportunities for marketers and brands, they will also usher in a host of challenges. For example, new contractual arrangements concerning digital property will need to be both developed and policed. The ease of copying data on digital platforms may facilitate potential snowballing or unofficially replicated material flooding the markets. Likewise, young consumers might over purchase immaterial “gimmicks” that serve little purpose beyond digital status symbols. Though most expect the cost of virtual goods to be a fraction of what physical goods cost, this is not always the case. For example, a virtual Gucci handbag recently sold for $4,115 while the physical bag retails at $3,400 (Ernest, 2021). Ultimately, conspicuous consumption and materialism, which typically deteriorate rather than increase consumer well-being (Dittmar et al., 2014), may be accelerated through the technological catalysts to be found in a future metaverse. Furthermore, transactions in the metaverse will likely be based on crypto currencies. While several challenges of crypto currencies have been solved (e.g., high energy consumption for mining), the underlying principles (blockchain technology, fluctuations etc.) remain quite complex, and platforms often lack mechanisms to protect consumers (Trautman, 2022). These aspects may be particularly crucial for young and less tech-savvy consumers who might use these platforms only to follow norms dictated by group pressure and status seeking.

Metaverse providers may address these issues by implementing and policing strict age restrictions, developing user friendly interfaces, and the application of clear consumer protection rules (e.g., return policies). The implementation of these measures can be challenging, especially if metaverse platforms are decentralized and/or located in countries with less developed political and economic regulatory systems. However, we argue that the most promising approach to these issues is to educate societies so that they may have a deep understanding of metaverse market mechanisms.

#### New Challenges of Privacy

Privacy is the primary issue surrounding both legacy social media and burgeoning metaverse applications. The most common issues center around the threat of sharing personal information with powerful entities, including brands. The marketers managing these brands can, through monitoring behaviors and brand sentiment exhibited online, determine when an individual is most likely to respond to particular types of marketing messages (Liu et al., 2020). The dynamic nature of these digital spaces allows for an unbroken chain of A/B testing that may allow marketers to fine tune their messages to increasingly manipulate individuals. More importantly, characteristics of those interacting in digital spaces can be extrapolated to others who might avoid these technologies. If this is an issue in legacy social media, it has potentially both deeper and broader impacts in metaverse applications. Many XR technologies that are required to access the metaverse continuously collect information about users, such as eye tracking cataloging offline focal points. AI algorithms are so powerful that they can use data collected in a device’s inertial measurement unit (IMU) and controllers to estimate the movement of currently untrackable body parts, such as legs (Winkler et al., 2022). Likewise, many XR devices make use of tracking technology to improve the user experience, such as through foveated rendering (a technique that uses eye tracking information to decrease rendering workload by reducing the resolution and sharpness in the peripheral vision area). Furthermore, inside-out tracking technologies in VR can generate information about a user’s physical environment, which is typically one’s personal space. The amount of information gathered from such devices goes way beyond what is possible with social media technology that tracks “only” digital variables.

With AR, the potential challenges increase even further. AR devices are based on a detailed understanding of a user and their physical context (von der Au et al., 2023). In contrast to VR devices, AR glasses are often superior in terms of tracking capacity (e.g., multiple cameras and depth-scanners, LIDAR, etc.). Since AR glasses can be worn in virtually any situation, the potential threat to a user’s privacy is extended to the privacy of other people who surround the user (Rauschnabel et al., 2018).

Both hardware and software offer potential approaches to address privacy issues. Similar to dash cams in cars that store information only for a limited time and overwrite information that is not accessed by a certain date, metaverse applications may be designed to wipe all consumer data after a relatively brief time period. Hardware producers might also ban facial recognition or implement automated anonymization technologies. However, these measures should be balanced against the loss of functionality that might accompany them. Regardless of what the designers do, tech savvy users and hackers will eventually find ways to bypass these mechanisms. Similarly, cities might ban the use of XR technologies in public spaces which would stall XR development. Furthermore, such bans might be challenging to implement, especially since tracking devices are getting smaller and AR glasses can be virtually indistinguishable from regular glasses.

#### Challenges of Diminished Reality

Legacy media and social media can impact our perception of reality only by censoring what they cover/report and, as a result, what we perceive. Metaverse technology can actively blot out information from the real world that we might find offensive or otherwise undesirable. In particular, “diminished reality” is a specific form of AR that estimates an object’s background and overlays data to blot the object out of the user’s view (Rauschnabel et al., 2022b). Diminished reality can eliminate physical objects from the user’s perception of the real-world, even though the product remains in the physical world. On the positive side, consumers might appreciate ad blockers that use diminished reality to blot out annoying signage.

**Fig. 2:**
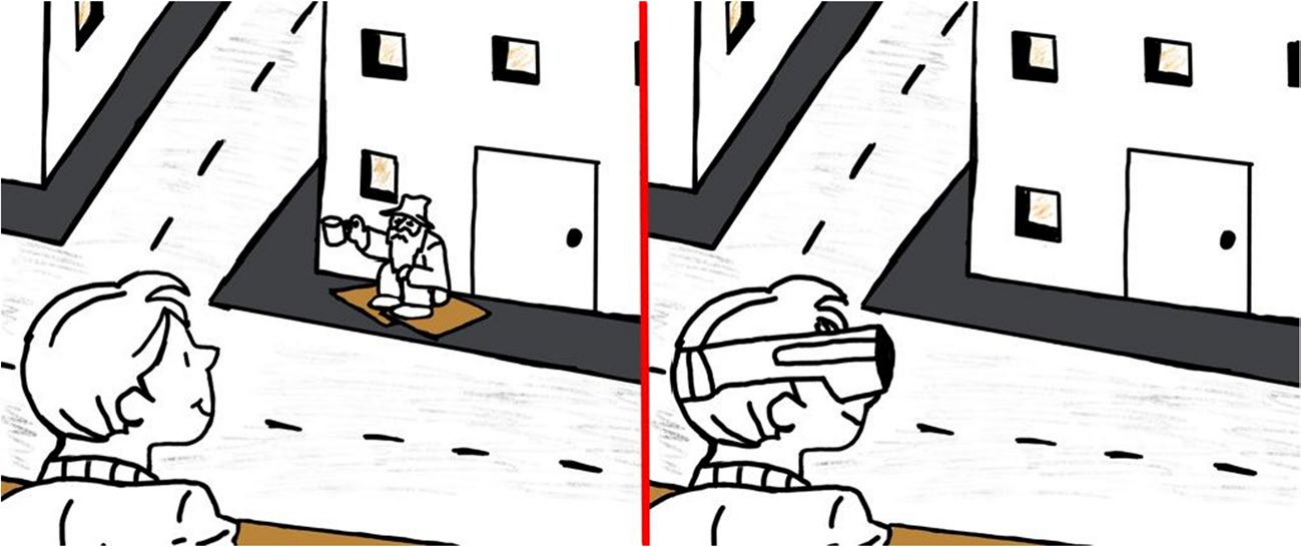
Removing real world content through diminished reality (Illustration: Annika Pezold, UniBw)

However, diminished reality technology can also raise concerns. Elimination of physical objects from a user’s perception may result in collisions. Furthermore, the entity that controls the ability to diminish reality can also be a cause of concern. Hackers, legacy media, or government entities might secretly install diminished reality applications on AR smart glasses to alter what some individuals perceive in an effort to manipulate the user’s attitudes or behaviors. However, users themselves might also use diminished reality unethically. For example, homelessness has become a significant issue in many major cities, and the newly elected mayor of Los Angeles, California, has issued an emergency declaration to allow for greater flexibility in using public funds to address the problem. Diminished reality could be used to overwrite objectionable content so that users do not even perceive the presence of homeless individuals. Figure 2 shows that a homeless person can be excluded from the user’s view allowing for a more “pleasant” perception of the environment (Fig. 2).

Overall, we contend that diminishing reality may prove to be one of the most pressing challenges associated with the emerging metaverse. Preventing intentional unethical use initiated by the user poses a more difficult problem. Terms of use may prove effective with many users, though savvy users will almost certainly find workarounds to these issues. As to the possibility that hackers, government entities, or legacy media may find a way to manipulate the user’s perception through this technology, these solutions are yet to be discovered.

#### A Question about Regulations

How should policy makers react to these challenges? There is a spectrum ranging from “laissez fair” to strict regulations and prohibitions, but each has its own set of issues. Many countries need to determine their desired position on this scale. For instance, the European Union currently has legal frameworks including Digital Rights Act (DRA), Digital Service Act (DSA) or General Data Protection Regulation (GDPR) that provide generic legal boundaries applicable to today’s metaverse-like platforms; however, adjustments might be needed once true metaverse platforms emerge. Furthermore, too much regulation may kill innovation in the metaverse context. The metaverse is rapidly evolving and it is anyone’s guess what this format will look like in the coming years. Depending on the characteristics designed into each use case and the evolution of the underlying technologies (e.g., blockchain), early-stage regulations stand a good chance of either not impacting the variables that drive adoption or stifling forward progress. We argue that political interventions, if any, should proactively focus on supporting the development of metaverse technologies as these technologies mature. Academics and technology pioneers are still struggling to understand exactly what the metaverse is and how it will look like in the future. Cross-disciplinary research collaborations as well as firm sponsored R&D projects will drastically expand what we know about this medium. Prospective users must understand what the metaverse is, how they can benefit from it, and how they can reduce the risks associated with participation in this rapidly emerging domain.

#### Limitations and Future Research

The paragraphs above discussed three significant challenges facing the metaverse: Privacy, diminished reality, and the true value of virtual objects. We acknowledge that this list is not exhaustive, and topics such as cyber mobbing, metaverse addiction, or the threats associated with the complexity of the technology (e.g., underlying blockchain technology) have not been addressed. We call for future research to systematically assess the dark side of the metaverse. Such findings should be used to design frameworks for a better metaverse future that benefits, consumers, firms, and society as a whole.

### Human – Computer Interface in Metaverse: Perspectives from Dark Side – Satinder Juneja, Preeti Tak & Ashish Gupta

The term "Metaverse" first appeared in the classic science fiction book "Snow Crash" written by Neal Stephenson in 1992, wherein he conceptualised "metaverse" as a community of virtual, lifelike avatars that assemble in true-to-life 3D virtual reality settings (Joshua, 2017; Dwivedi et al., 2022a, 2023a). Informally defined as the meeting point between the physical and virtual worlds, the metaverse is still partially in the realm of speculation because it is in its early stages and has not been fully defined (Ball, 2022). Given the steep proliferation of technology in our lives, we live in a network. Be it communication, e.g., telephony, text message exchange (WhatsApp or SMS), participating in social media or going about doing usual tasks, e.g., paying by credit card, entering the airport by QR code scan of our boarding pass, writing, and sharing on blogs. We are leaving multiple digital traces for each interaction and more. Gartner (2022) predicted that 25% of users would spend at least one hour per day in the metaverse by 2026. The scale and speed with which this data is being amassed and interlinked have given rise to computational social science. The metaverse, given its base, is deeply rooted in enabling technologies such as blockchain, augmented reality, virtual reality, etc., and would rely heavily on Human–Computer Interface (HCI). The extant literature has discussed the seamless opportunities which Metaverse offers to the business to engage and reach consumers in different domains such as fashion, retail, tourism, hospitality, advertising, branding etc. (Dwivedi et al., 2022a, 2023a; Hollensen et al., 2022; Kim, 2021; Joy et al., 2022; Buhalis et al., 2022; Gursoy et al., 2022) and consumers. Despite the many opportunities that the metaverse provides, an increasing number of incidents show that the platform undoubtedly has a "dark side".

The infrastructure that supports the metaverse, comprising both traditional Information Technology (IT) and Operational Technology (OT), comprises a wide range of technologies that manage component sensing, physical interconnections, and interactions. While most IT protocols can be made secure, there are no design guidelines for information security and privacy in OT. Bad actors will therefore be able to thwart commercial transactions by stealing or changing the goods, the service, or the intellectual property (IP), stealing or diverting the buyer's funds, eavesdropping, or manipulating the transactions that may be happening between a seller and a buyer.

#### Dark Side in Metaverse

The following are potential areas of the dark side in the metaverse:

##### Spoofing and Phishing

To navigate the metaverse, everyone has to create virtual avatars. As the platform is still evolving, there are instances of creating convincing 3D representations of real-world brands/products/humans, which makes identity protection essential. However, the metaverse has failed to comply with the same, and in May 2022, a phishing scam was reported on Decentraland, a popular Ethereum-based virtual world (World Economic Forum, 2022a). The scammers were able to create a fake website that was similar to the Decentraland website. The websites then tricked users into inputting their private keys, allowing the scammers to steal their cryptocurrency. With few regulations and less familiarity with platforms, the consumers and the brands are at risk (Pooyandeh et al., 2022).

##### Privacy Concerns

The metaverse might collect and collate users' personal information, including eye-tracking, physical reactions, and haptics (Fernandez & Hui, 2022; Hirsch, 2022). Due to the immersion, the indistinguishability of virtual and reality, and the multi-sensory nature of the metaverse, more specific user data will be gathered than on earlier platforms like social networks, which increases the interest of unethical parties in this information, increasing the risk of network attacks (Leenes, 2008; Zhao et al., 2022).

##### Cybercrime & Laws (Drug trafficking, terrorism, and other illicit business)

The metaverse's regulatory ambiguities will need to be identified and resolved (World Economic Forum, 2022b). For example, there is no clarity on when is a virtual act criminal in nature? Today on the internet, shadowy users can access covert services via the "dark web" a secretive environment, and there is much illicit trade and terrorist-aiding activities are being held there, away from the prying eyes of authorities (Chertoff, 2017; Kaur & Randhawa, 2020; Weimann, 2016). The sheer scale, experiential dimension, coupled with anonymous nature of the metaverse would enhance this issue to altogether a different level.

##### Inequality in Metaverse and rise of Digital Divide

How can we promise that everyone has access to the handsets, headsets, and connectivity required to participate in the metaverse? While the benefit of access means participation in all societal domains, the lack of it would alienate the deprived ones from such participation in societal domains and can potentially impair societal development (Van Dijk, 2017). The impact of digital access and divide was visible during COVID-19 (Ramsetty & Adams, 2020), which shows that digital access can provide access to better benefits for various sections of society.

##### Gambling

Gambling is popular and easy in the metaverse, where minors can gamble even with age restrictions. Rosen (2022) reported that the ICE Poker Virtual Casino has made over $7.5 million in the previous three months and listed as the high daily traffic to Decentraland (a popular metaverse location). As users gamble tokens rather than actual money, the lawsuits against them are frequently dismissed, thus, leading to duping/cheating of gullible users.

##### Vulnerable and Impressionable Children and the Effect

How do we keep an eye on what children are doing and seeing as they immerse themselves in the metaverse, so that we can keep them safe? Children are highly prone to implicit and explicit cues and peer pressure. The scale, attractiveness (sound, imagery, virtual, 3D), and inquisitiveness of the metaverse are bound to draw children to it. Several adolescents committed suicide in different countries because the video game "Blue Whale challenge" shows the havoc internet can have on the lives of this impressionable population (Lupariello et al., 2018). Given the avatar concept and ability to quickly change due to digital elements, the threat of cyberbullying can also have a larger impact on children in the era of the metaverse (Wong-Lo et al., 2011). In December, Hayward (2022) reported that Epic Games was penalised $520 million in settlements by U.S. Federal Trade Commission (FTC) which involves a $245 million refund to impacted customers by the FTC for what it deems "dark patterns" and "billing practises," as well as a $275 million fine that will be paid to the agency for breaking the Children's Online Privacy Protection Act (COPPA).The platform collected personal data from children under age 13 without notifying the parents. Additionally, Epic deceived the gamers into unintentionally spending hundreds of millions of dollars on in-game purchases by using "counterintuitive, inconsistent, and confusing button configuration" (Hayward, 2022).

##### Negative Impact of Health and Possible Addiction

How can we tackle the health risks of an immersive environment where VR hangovers, post-VR sorrow, and cyber addiction are real? International population studies have shown that internet addiction is proliferating quickly and is becoming more widespread globally. Smartphone use is dramatically expanding, and social networking sites are becoming increasingly popular, leading to psychological risk factors (Chertoff, 2017).

##### Anti-Social Behaviour

The metaverse is conceptually based on avatars which means one could have different avatars from the actual person, which could be very different from the 'real-verse' (Glavish, 2022). The anonymity or personification allowed due to this avatar may encourage some unscrupulous actors to exhibit dark and uncivil behaviour towards other strata of society. As per Non-profit Center for Countering Digital Hate, within the VRChat platform, a violating incident, occurs about once every seven minutes (Frenkel & Browning, 2021). The metaverse can encourage latent dark/uncivil behaviour of human beings due to obscure/ill-defined digital personalities. They may not exhibit such behaviour when they fear societal norms related to a persons’ identification.

##### Pornographic Content

The pornographic content available on the metaverse may be categorised into two parts: Sexually explicit material and sexually explicit activity. The BBC, in February 2022, reported that anyone older than 13 could enter a strip club in the metaverse through two main routes: VRChat and Roblox, thus exposing children to sexually explicit material. The metaverse also threatens children by exposing them to sexually explicit activity. Furthermore, using an app with a minimum age rating of 13—they visited virtual-reality rooms where avatars were simulating sex and she was shown sex toys and condoms and approached by numerous adult men.

##### Reduced Sensitivity and Empathy

Due to the primary nature of interaction in the metaverse being digital, there is a possibility that people may lose or have reduced sensitivity to others when in Metaverse. How can we prevent individuals from being desensitised to violence, racism, and misogyny as they immerse themselves in increasingly realistic virtual worlds? Recently a woman researcher reported rape in the metaverse reporting that her avatar was raped and abused while other users watched after he led her to a private room at a party on the Horizon Worlds platform. Her experience shows a lack of decency and sensitivity from onlookers in the metaverse (Newslanes, 2022).

##### Interface Interference

Gray et al. (2018) defines interface-interference as any manipulation of the user interface that privileges specific actions over others, confusing the user or limiting the discoverability of important action possibilities. In the Metaverse, the enhanced intensity of user experience is also likely to accentuate interface interference and thus enhance the scope of manipulation and deception without the user's knowledge (Hong & He, 2021). Key components of interface interference are hidden information, preselection and aesthetic manipulation. The Federal Trade Commission asserts that consumers were charged in the most recent Epic games penalty case even if they were waiting at a loading screen or attempting to restart the game from sleep mode with a single button click. Users allegedly lost hundreds of millions of dollars as a result of these dubious tactics, according to the complaint. According to allegations, Epic disregarded more than a million user complaints, leading many consumers to believe they were being overcharged. The $245 million compensation from Epic will now be utilised to reimburse consumers who suffered financial losses.

#### Conclusion

As the hype around the metaverse continues to grow, it is important to understand and address these issues about the 'dark side'. There are several antecedents of these dark side elements. Firstly, consumers lack the rationality to use metaverse-like platforms more positively. The platform is created to enhance quality interaction, but it may lead to negative experiences if not handled maturely. Secondly, large-scale moderation of content is very difficult. For instance, Facebook deals with three million posts requiring daily moderation, which may lead to confusion, hate crimes, etc. Lastly, the age verification measures are easy to circumvent, thus exposing children to a plethora of harmful content which may be detrimental to their growth. Metaverse was conceptualised to revolutionise how people connect, socialise, and enjoy their leisure time, but like any breakthrough that creates new possibilities, it also makes room for the shadowy aspects of human nature.

### A Study of the Potential Dark Sides of the Metaverse in order to Make Immersive Experiences More Secure–1Sanjar Mohamed AB

The metaverse is the next generation internet—a shift from 2 to 3D ways of consuming information by seamlessly integrating with our physical and virtual lives to create real-time immersive experiences. The development of the metaverse will be bolstered by the advancement and adoption of essential technologies, including 5G communication, extended reality (AR/VR), artificial intelligence, spatial computing, blockchain, and digital twins.

In the metaverse, we will live, connect, play, learn, earn, and work in a fundamentally new way. In essence, the metaverse humanizes technology by allowing humans to interact and perceive the digital world.

In the holistic and democratic vision, the metaverse is a collection of interoperable, decentralized, spatial and persistent immersive virtual worlds that allow people to take control of their data and digital assets. This means people can move their avatars and digital assets such as clothes and collectibles from one virtual world to another.

The metaverse is still in its infancy, and there are many questions and concerns regarding user protection and criminal activities. History has shown humanity that the advent of new technologies will provide new attack vectors for bad actors. Question: Is there much we have learned from history?

#### Learning from the Past

The first evolution of the internet has created the dark web. All kinds of criminal activities are carried out on the dark web, such as the sale of weapons, drugs, exploitation of children, and pornography. In the absence of proper guard rails and regulations, the volume, impact, and implications of criminal and illegal activities in the metaverse could be horrendous. By monetizing user attention and data, web2 architecture has also allowed companies to build walled gardens and amass wealth, violating user privacy. In addition, political parties, propagandists, and governments have used social media platforms to spread misinformation, disinformation, and propaganda to manipulate public sentiment and decisions.

Metaverse adoption will only exacerbate the problems of the previous generation of internet and web without government regulation and responsible approaches from organizations. Also, institutional investors and venture capitalists should fund immersive virtual world projects that support sustainable and safe metaverses.

An understanding of the potential problems from a holistic and futuristic perspective is crucial to addressing the issue. As with the early days of the internet, we cannot be sure that all adverse effects of metaverse and cybercrimes will occur in the immersive world. Researching metaverse impact and developing policies to ensure collective ownership is essential to developing a safer metaverse. If proper regulations are not in place, there might be problems in the metaverse.

#### Data Harnessing and Identity Theft

Metaverse users will generate a huge amount of information (big data) when they engage in immersive worlds, especially if they use AR/VR devices. In the metaverse, companies can capture invasive personal information about users through face, eye, haptics, and sensors. Data can include blood pressure, breathing rate, facial expression, vocal inflection, eye movement, body movement, etc. Using more advanced systems such as brain-computer interfaces (BCI), organisations can also collect deeper and thicker data. The metaverse maps detailed biometric information and comprehensive online identities (Big Think, 2022a).

A paper presented by researchers at Meta reveals that when processing from just a few sensors on the head and hands of the user, AI could accurately predict the posture and motion of the user (Winkler et al., 2022)*.* Metaverse identities include avatars, biometrics, spatial information, conscious and unconscious information generated by interaction with platforms. These data can provide insights into an individual's desires and mannerisms. Data extraction and exploitation for invasive marketing or political purposes pose a problem.

What happens if bad actors are able to access a person's geospatial and biometric data? Criminals extract data and steal the identity of the user. By using deep fake technology, criminals can impersonate a particular user without being identified. In the metaverse, identity theft is a major concern.

#### Invasive Advertising, Misinformation and propaganda

Virtual objects can be placed in the metaverse as promotional elements in the natural environment of virtual worlds. Users subconsciously perceive these virtual objects as natural environments, when in reality they are products placed intentionally based on a user's emotional and behavioural profile. A more aggressive marketing strategy would be to place marketing AI avatars within the metaverse. Marketing AI avatars are indistinguishable from other avatars and can interact with users as they would with any other avatar. Marketing AI avatars can personalize the content of marketing in the most persuasive way because they have a complete profile of the user and can't process emotions based on facial expressions and vocal inflections.

Dr Louis Rosenberg, CEO of Unanimous AI, believes that unregulated metaverses will challenge the notion of free will and serve as a weapon of mind control (Big Think, 2022b). A more dangerous scenario is when AI avatars are used to spread propaganda, misinformation, and disinformation. Governments and political parties can aggressively use this. Data can also be used to discriminate against certain races, regions, and religions. By tailoring their message to appeal to the people inclined to their ideologies, terrorists and extremist groups can use the metaverse as a recruiting tool.

#### Phishing

The metaverse can also be used by criminals to conduct phishing attacks. It is a major security concern that bad actors can create metaverse representations of real-world brands and products. It is possible for users to be tricked into giving away their personal information or sending cryptocurrency or tokens to counterfeit brands and products. According to Chainalysis, phishing scams are on the rise in the metaverse (CNBC, 2022a). The Ethereum-based metaverse Decentraland was recently targeted by a phishing scam. Scammers set up fake Decentraland websites. By tricking users into entering their private keys, scammers gained access to the assets and stole cryptocurrency.

#### Financial Crimes and Terrorist Activities

The metaverse can become a hotbed for financial crimes if unmonitored and unregulated (Elliptic Metaverse Report, 2022). Metaverse platforms use cryptocurrencies and on-chain tokens to make transactions within the metaverse. This means selling and purchasing of digital assets provides opportunities for cross border financial transactions in a manner that is more difficult for the authorities to monitor. Cybercriminals use cryptocurrency to launder money and commit other crimes. Researchers at the National Counterterrorism Innovation, Technology, and Education Center in Omaha say the metaverse could be used by terrorists as a new domain (Elson et al., 2022). There is a possibility that terrorist groups will use metaverse virtual currencies to make transactions. For these issues to be addressed, international and national law enforcement agencies must work together.

According to Hayward (2022), Epic Games was penalized $520 million by the U.S. Federal Trade Commission (FTC) in December. For alleged "dark patterns" and "billing practices," the FTC is refunding $245 million to affected customers, and they are fined $275 million for violating the Children's Online Privacy Protection Act (COPPA). Children under the age of 13 were collected by the platform without their parents being notified (Hayward, 2022).

#### Abuse of Children, Harassment of Women, and Pornography

Protecting children and women in the metaverse is a priority. A BBC news researcher posing as a 13-year-old, accessed the VRChat platform and was allowed entry to virtual strip clubs and witnessed grooming, sexual material, racist insults and a rape threat in the metaverse like world (BBC, 2022). The researcher was shown sex toys and condoms, and approached by numerous adult men. These incidents show the gravity of sexual attack, exploitation and abuse and children might have to face an unregulated metaverse. When more and more users engage with metaverse, new forms of harassment, abuse and discrimination will emerge. In the Roblox platform, people create sex ‘condos’ to talk about sex and make their avatars have virtual sex. This definitely goes against the policy of Roblox. In 2007, a woman reported that she was virtually raped—in Second Life (Wired, 2007).

#### Call to Action—Regulations and Responsible management

A group of researchers appointed by the French government has advised leaders to start regulating the metaverse and not repeat the mistakes made with social media (CoinDesk, 2022). There are many challenges associated with policing the metaverse. A growing number of companies and startups are building metaverse worlds. Law enforcement agencies will have a huge task keeping track of all the metaverse worlds continuously. It is difficult to review the veracity of an incident because every metaverse user will have their own version of events, and interactions do not leave digital footprints as they do on social media. In order to design policies, take proactive measures, and monitor metaverse laws, law enforcement will need highly qualified and specialized personnel.

The metaverse will have stalkers, cybercriminals, abusers, frauds etc. Some of the potential dark themes of the metaverse include identity theft, financial fraud, organised crime, extremist and terrorist activities, human trafficking, pornography, harassment, and sexual assault.

However, the metaverse does not demonize technology, the metaverse will enhance human experiences and connections. It will also change the way we learn and work. The danger lies in how bad actors could use the metaverse for criminal, obscene, and illegal activities. A sustainable metaverse world can be built through proper regulation and coordinated government efforts to build a safe, inclusive, and transparent immersive internet.

### The Impact of Metaverses on Social Inclusion and Wellbeing—Savvas Papagiannidis

Over the past two decades, the proliferation of digital technologies has had a significant impact on the business and social environment, leading to the emergence of electronic space and new geography (Li et al., 2010). Metaverses promised to extend the choice users have when it comes to electronic spaces not just quantitatively, but also qualitatively. Free from the burdens of the physical world, metaverses could enhance social inclusion opportunities by opening access to diverse spaces and communities in an immersive manner (Papagiannidis et al., 2008). Digitally transporting users to different worlds is a challenging proposition, though, and is likely to remain so for the foreseeable future. In the physical world, Burchardt et al (1999) consider individuals as socially excluded if they are geographically resident in a society, cannot participate in the normal activities of citizens in that society, and would like to participate, but are prevented from doing so by factors beyond their control. Compared to typical web-based solutions, accessing metaverses can be a more complex process that requires more ICT resources. Similarly to how physical transportation from one space to another may require a car or public transport, entering the metaverse can require specialised hardware and premium services that may not be possible to reuse across metaverses due to interoperability issues. So, access can be an issue before one can actually enter the metaverse. Even if one had access to the resources required and had the skills to operate such devices, wearing headsets for a long period of time can be tiring. Also, unless hardware designs consider more actively diverse groups of users who may have very different accessibility needs, many users could be left out. It is important to consider designing accessible and inclusive technologies that can underpin immersive environments as they empower users with new information and experiences without the limitations pertaining to the physical environment (Zallio & Clarkson, 2022). It is an oxymoron that the technologies and designs that promise to lift the limitations of the physical environment can impose obstacles of their own.

In addition to the above, the synchronous and involved nature of interactions in metaverses means that temporal barriers can also limit participation. Social inclusion is not just a matter of overcoming constraints of space, but also overcoming these constraints at particular moments of time so as to gain access to the informal networks of work, leisure, friendship, and family (Cass et al., 2005). This space–time access challenge can be further exacerbated by the limitations that having to use specialised hardware poses. Even if one had such hardware, one could only join a metaverse from the location where the hardware was available. Such “metaverse terminals” may be opening up new worlds, but only from specific physical locations, effectively limiting users. The alternative would be for users to carry such hardware with them, which is likely to be a major inconvenience, if at all possible.

Of course, on one hand it could be argued that hardware will improve over time and such limitations will be eventually addressed. Hardware will also become more affordable. Although the above may be true, such developments are likely to take many years. Providers could focus on early adopters and create worlds that focus on them as opposed to the majority of the potential users in the long term. Similarly, from the user end, during such a period metaverse spaces and communities will be shaped only by those who could actually access them. Those excluded via other channels, who could have found an alternative inclusion route to communities and activities of interest, will remain excluded. Effectively, a technological breakthrough that could have contributed to reducing exclusion could potentially exacerbate existing (or create new forms of) social exclusion (Andrade & Doolin, 2016). On the other hand one could also argue that specialised hardware is not always required for accessing metaverses. In some cases, access can be achieved by using a web browser on personal computers or via an app on mobile phones. Such an approach could in fact lower access barriers. Still, the experience would be radically different. If one were to reverse and push the argument and consider simpler ways of accessing metaverses that sacrifice three-dimensional immersion in favour of access and participation, then why do we need metaverses to start with? One could use the analogy between having to use a slow versus vs a fast broadband connection to access the Internet to put this in perspective. The user can still access the service, but the experience can be very different.

*Considering the above, research will need to revisit adoption and acceptance issues of metaverses from different user group perspectives, taking into consideration their different characteristics and requirements. Such research could pave the way for understanding factors that impact digital inclusion and help identify opportunities for tackling new manifestations of the digital divide and poverty.*

When referring to online technologies (such as social media or online gaming) with regards to wellbeing, discussions often revolve around addiction. This is surprising as wellbeing, as a broader concept, is not related to the absence of an undesirable state (Vanden Abeele, 2021). As such it is important to consider digital wellbeing more widely. To this end, Vanden Abeele (2021, p. 938) defines digital wellbeing as “^2022^, p. 5). Such circumstances are not new, and being active on social networking sites can have such an impact on users. Being immersed in metaverses and having direct interactions that go beyond written forms of abuse can take things to a completely new level. Metaverses not only inherit many of the dark side aspects of social media (Baccarella et al., 2018*a subjective individual experience of optimal balance between the benefits and drawbacks obtained from [mobile] connectivity. This experiential state is comprised of affective and cognitive appraisals of the integration of digital connectivity into ordinary life.*” The immersion and flow that metaverses can develop may make finding a balance between experiencing maximal controlled pleasure and functional support, while maintaining control and avoiding functional impairment, a challenging one. In fact, such a balance can be easily disrupted by exogenous factors that are beyond a user’s remit. Unlike most Internet applications that users have experienced, such as web browsing or shopping online, metaverses are supposed to be social spaces that encourage direct user-to-user interaction. *“The excessive noise, overwhelming information, behavioural, verbal or visual harassment can lead to a variety of emotions such as fatigue, depression or embarrassment, which in turn could lead to a loss of self-confidence or even consequences of greater impact for the user.”* (Zallio & Clarkson, ), but extend and magnify them.

*Given the above, it is important to not just study the factors that positively and negatively affect digital wellbeing and in turn how this affects a user’s overall wellbeing. Such studies could consider different user segments, paying special attention to potentially vulnerable groups.*

To make matters worse there are limited ways one can protect one’s avatar and actual self from such an effect. User behaviours in metaverses can be difficult to monitor and regulate and consequently there is often little to prevent users from acting in an offensive manner. Even defining “anti-social” can be problematic as the ethics and norms applying in virtual worlds are not necessarily the same as those in the real one (Papagiannidis et al, 2008). Such behaviour can be detrimental to one’s digital wellbeing and potentially their overall wellbeing. Virtual worlds may be virtual, but actions in them can have very real and tangible consequences. This is why it is often more appropriate to refer to virtual worlds as synthetic worlds, in order to highlight the fact that these worlds are products of human actions (Malaby, 2006). Such connections to the real world should not be treated as an attempt to stifle freedom of expression and to break the “*magic circle*”, but as a way of acknowledging that the two need to exist harmoniously. Castronova (2005, p.147) considers the boundary between the synthetic world and real one to be a porous membrane: “^2022^; Bojic, 2022*we find human society on either side of the membrane, and since society is the ultimate locus of validation for all of our important shared notions – value, fact, emotion, meaning – we will find shared notions on either side as well*”. Still, on the synthetic side the rules are created and enforced by the companies that created the metaverse and have a commercial interest in maintaining it. Whether such interests are aligned with user interests is open for debate. Social media platforms are testimony to the challenges companies and users face when it comes to issues such as freedom of speech, identity management or harassment. This is not to say that tech-companies should not seek commercial success. Quite the contrary. This is important as otherwise no metaverse could be sustainable. It is a statement of the need to identify ways in which financial sustainability and success will not be achieved at the expense of the wellbeing of individuals and communities. Just as it is often the case that the companies encourage users to co-create by creating the content they enjoy in metaverses they should also consider involving them in the governing and shaping of the worlds themselves, including deciding how porous the magic circle should be.

*Research should pay special attention to consequences and outcomes that go beyond the metaverse boundaries and can spill over to real-life.*

*Preventative measures need to be sought that would make it possible to proactively act as a first line of user defence from such behaviours without, though, compromising user privacy and experience.*

### Darkside of Metaverse: Mental Health and Wellbeing Perspective – Alexandra Taylor, M. Claudia tom Dieck, and Timothy Jung

#### Possible Negative Aspects of the Metaverse

Despite its potential benefits, it is essential that research discusses the potential dark side of the metaverse, regarding consumer mental wellbeing. Utilising our knowledge pertaining to older media sources and the technologies involved within the metaverse, we can begin to explore its potential detriments. For instance, research has already warned of the addictive use of the metaverse (Barreda-Angeles & Hartmann, ); highlighting motivation and psychological reward as dictating variables responsible for level of addiction (Barreda-Angeles & Hartmann, 2022; Hussain et al., 2018). Unfortunately, the severity of technological addiction is often depreciated by society and thus its consequences go unaddressed (Grajek et al., 2022).

Left unchallenged addiction can instigate negative mood modification (Barreda-Angeles & Hartmann, 2022). Similarly, motivation has been highlighted as a key factor in determining positive/ negative mood (Javornik et al., 2022). This is especially true for users who engage in self-indulgent escapism, as this can trigger anxiety, depression, and aggression within a user’s objective life (Grajek et al., 2022; Panova & Lleras, 2016).

Consistent with clinical health, additional evidence depicts how virtual reality (VR) elicits depersonalisation and derealisation (DPDR) (Aardema et al., 2010; Peckmann et al., 2022). As users express feeling detached from their actions, thoughts, and body, and begin to doubt reality. Persistent DPDR can adversely affect a person’s functioning and is often concurrent alongside depression and anxiety (Michal et al., 2016; Peckmann et al., 2022). Again, demonstrating how the metaverse can impact a user’s objective mental wellbeing.

Due to the highly social nature of the metaverse, it is also wise to consider the behaviours of its users. Research reports increases in anti-social behaviours such as cyberbullying and harassment (Henz, 2022; Qasem et al., 2022; Wiederhold, 2022a, b). Wiederhold ([Bibr CR204][Bibr CR205][Bibr CR124]) argues the effects of which are comparable to real-world incidents; due to VR’s ability to induce real anger and sadness, which is then carried through into the real-world (Kothgassner et al., ).

#### Future consequences

Due to rapid brain development, children will disproportionately experience the long-term impacts of the metaverse (Grajek et al., 2022; Kaimara et al., 2021). Predominantly, these concerns consider the psycho-social aspects of wellbeing; relating to addiction, anti-social behaviours, isolation, and emotional development (Kaimara et al., 2021). Already there is evidence of increased ADHD (Attention Deficit Hyperactivity Disorder)-like behaviours manifesting in young metaverse users, demonstrating the metaverse’s influence on childhood development (Steve & Grubb, 2018).

Moreover, overuse of these technologies reason apprehension relating to a potential increase of isolative behaviours (Kaimara et al., 2021). As the metaverse expands we will see less human-to-human connection and more human-to-artificial intelligence (AI) connection (Henz, 2022). This phenomenon will propose a new form of loneliness as the introduction of AI risks alienating consumers (Putoni et al, 2020).

Simultaneously, the introduction of AI within the metaverse will allow easier access to user-related information (Henz, 2022). The more information accessed via AI; the more consumers will become vulnerable to the manipulation by the organisations attaining their information (Henz, 2022). These manipulations will limit self-control over mental experiences and behaviour, thus wellbeing (Yaden et al., 2018).

Despite increased accessibility to certain services provide by VR, the technology alone remains costly to develop and purchase (Yaden et al., 2018). Thus, the metaverse, is more acquirable for affluent populations, than those faced with economic inequalities (Riches et al., 2021; Usmani et al., 2022a, b). This chances the perpetuation of the socio-economic digital divide and thus the social exclusion and discrimination of certain demographics (i.e. the elderly) (Yaden et al., 2018). Consequently, these demographics will forgo any wellbeing-related benefits provided via the metaverse (Usmani et al., 2022a, b).

#### Mitigation-Strategies

As the metaverse expands, it is important that stakeholders and researchers recognise and educate themselves of its associated risks and benefits (Wiederhold, 2022a, b; Yaden et al., 2018). Acquiring the knowledge and advice of healthcare professionals will ensure policy and guidelines are ethically developed in accordance with consumer wellbeing (Wiederhold, 2022a, b; Yaden et al., 2018).

Adopting professional perspectives will additionally inform the governance of the metaverse and subsequently its users. Governance will help mitigate anti-social behaviours such as racism, discrimination, and bullying (Dwivedi et al., 2022a). *Governance by the metaverse* will ensure adherence to terms of service, by holding users accountable for their behaviour—thus incentivising positive socialisation among its users (2022a).

Preliminary evidence indicates that duration of use and feelings of embodiment are predictive of unfavourable addictive behaviours (Barreda-Angeles & Hartmann, 2022). Therefore, to safeguard consumer mental health and minimise the risk of addiction, derealisation, and social isolation, policy should propose time limits.

Additionally, developers should aim to limit interference of the digital divide by standardising technological cost and its provision. In terms of wellbeing service, the metaverse should be treated no differently than existing resources (Yaden et al., 2018). Furthermore, vulnerable groups, such as the elderly, could benefit from educative VR sessions, which aim to minimise technology discrimination by overcoming usage and knowledge gaps (Li et al., 2021).

#### Research Agendas

Further research is necessary to strengthen the validity of novel insights currently available regarding the dark side of the metaverse (Wiederhold, 2022a, b). Previous literature highlighted the benefits of collaborating the perspectives of users, researchers, and professionals in ensuring ethical development (Wiederhold, 2022a, b; Yaden et al., 2018); this could be attained via a human-centred design (Fernandez & Hui, 2022). Currently, however, there is a lack of sufficient supporting evidence for this approach; thus, research should investigate the practicality in the development of a safe and ethical metaverse—which promotes consumer wellbeing.

The novelty of the metaverse means that minimal research is available which depicts the likelihood of negative risks associated with its use. Thus, justifying further investigation into the dark side of the metaverse; by expanding established knowledge regarding the overuse of existing medias such as internet addiction, depression, anxiety, social isolation (Dwivedi et al., 2022a). Findings will allow policy makers to prioritise guidelines in correspondence to the most prevalent risk factors associated with user wellbeing.

As illustrated previously, misconduct within virtual worlds can impact the mental wellbeing of those targeted (Kothgassner et al., 2017; Wiederhold, 2022a, b). This therefore amplifies the need for proper regulation and governance of the metaverse as suggested by Dwivedi et al. (2022a). However, imminent research needs to establish the feasibility of governing the metaverse and how this will be affected by differing local laws (Fernandez & Hui, 2022).

### Sexual Harassment in the metaverse: Will Extended Reality exacerbate toxicity online?—Animesh Jain & Richard Foster-Fletcher

XR refers to environments that combine the real and the virtual. VR, AR and mixed reality (MR) are all examples of this. This technology, while constantly evolving, is not necessarily new, nor are the opportunities and risks that come with it. When done correctly, XR can push reality to its limits, resulting in new experiences that, while artificial, fuel real and lasting memories. New XR developments make our virtual worlds more immersive. Furthermore, remarkable technologies make metaverse spaces feel more and more natural. Unfortunately, it's not just the surroundings that feel authentic; it's also the experiences. As a result, emotional reactions intensify in XR environments and can elicit the same internal nervous system and psychological responses as unpleasant real-life interactions. At first glance, XR may appear to be your typical sci-fi innovation, and it's easy to get caught up in the novelty. However, the true impact of these experiences is determined by how they make people feel. This makes them both valuable and dangerous because there are few rules, and it's hard to police these platforms effectively. By highlighting the issue of harassment in the metaverse, we will attempt to understand how the metaverse has the potential to exacerbate toxicity online.

#### XR Development to Elicit Emotions and Cases of Sexual Harassment

Organisations already use immersive technology to elicit emotion and motivate millions of people to take action and are investigating more ways to use VR's immersive technology to extend this further, highlighting the criticality in investigating what emotional reactions are experienced. The research by Durnell (2018) used the appraisal theory of emotion for interpreting the subject's emotional reactions, its sentiment and thematic analysis showed (a) an increase in empathy, (b) reports of emotional reactions, such as sadness, grief, and anger, (c) a better understanding of the crisis, (d) intentions to act in response to the crisis, (e) the importance of VR for educational use, and (f) the power of VR and its ability to change fields such as education, humanitarian work, and politics. According to this study, the immersive experience of viewing a crisis in VR elicits a wide range of highly emotional reactions. Studies have shown that more immersive VR settings had a more significant effect on fear and anxiety (Juan & Pérez, 2009) than on happiness and relaxation (Baños et al., 2008). Understanding the role of VR in eliciting emotional reactions and behavioural change is an important goal, especially given the rapid development of emotional VR content and people's access to immersive technology. VR is significantly more psychologically potent than other media due to the high levels of immersion and users tend to treat the VR experience at a cognitive level, as a real physical interaction.

For as long as there has been technology, there has also been the potential for its misuse. However, VR design makes it more difficult to escape the effects of cyberbullying. According to the research in Blackwell et al. (2019), "in VR, users are embodied in avatars that move when the player moves and interact with other players in 3D spaces, enabling violations of personal space and corporeal presence that feel fundamentally different than interactions that occur in other online environments—an experience made potentially more salient by the unique sensation of the presence or the feeling of truly *being there*. As a result, the embodiment and presence in VR intensify harassment." This feature of a virtual avatar in VR allows for the possibility of committing and experiencing virtual groping (Basu, 2021) and even rape (Smith, 2022), which was not possible in previous generations of online services or 2D spaces such as Instagram. Harassment can be exacerbated in immersive environments by features such as synchronous voice chat, increased feelings of presence and embodiment, and avatar movements that can feel like invasions of personal space (such as simulated touching or grabbing). Harassment and bullying may become more traumatising while protecting privacy will become even more important as data about an individual's digital location, emotional state, or behaviour becomes more accessible. Early reports of sexual harassment in virtual spaces highlight the critical need for proactive governance and regulation, particularly to protect vulnerable groups including children and protect their psychological well-being.

#### Governing the Metaverse

Harassment is common on the internet today, but being in the Metaverse can amplify it. Early indications from research and reports suggest that the technology companies in the space have a lot of work to do if they want to make the Metaverse a safer place for users than social media. As a result, this issue requires immediate attention. However, an important question remains: can a sexual harassment encounter in virtual reality be compared to one in the physical world?

In the real world, some people may sexually harass strangers and indecently expose themselves; the same is true in the metaverse. Indeed, this is more likely to occur precisely because legal authorities will find it difficult to hold them accountable. While criminals can use cyberspace to inflict harm and thus escape the constraints of the physical world, the ends they seek and the injuries they inflict remain grounded in physical reality. In VR, sexual harassment can be perpetrated in far more ways than was previously possible on traditional online platforms. There have been numerous reports of virtual groping and virtual rape. Federal prosecutors in Belgium (Wired, 2007) asked the Belgian Federal Computer Crime Unit to travel to the scene of a crime in the Second Life platform to investigate a "virtual rape" involving a Belgian victim. The reaction of Belgian police to a reported virtual rape suggests that such an experience is traumatic. As a result, it is critical to discuss the existing remedies for dealing with these new-age crimes. Accountability in virtual space becomes more challenging to define. Thus the availability of this new conceptual vector for human activity is not without flaws and has the potential to open Pandora's box of legal challenges. If much can be accomplished with images such as revenge porn, imagine what a sentient, 3-D avatar with a reach of billions of people could accomplish.

The distributed landscape of virtual reality applications and the dynamic nature of local community norms complicate efforts to govern these developing spaces. Authorities must better understand this nascent social and psychological environment in terms of harassment, abuse, and discomfort in social VR. Users' definitions of what constitutes online harassment are subjective and highly personal, platform- or application-level policies may be difficult to enforce. Furthermore, embodiment and presence in virtual reality spaces intensify harassment, while ephemerality and non-standardized application controls make it difficult to escape or report unwanted behaviour. While norm formation is especially difficult in virtual reality environments, and social VR has yet to develop shared norms for appropriate behaviour. So alternative community governance strategies are required for the metaverse to be prosperous and inclusive.

### Mitigation Strategies of Metaverse-trigged Unintended Consequences– Yichuan Wang & Carrie Yan

#### Unintended Consequences of Metaverse Utilization

Although the concept of the metaverse is still evolving and there is not yet a widely accepted definition, utilizing metaverse-related technologies (e.g., AR, VR, NFTs, and 3D reconstruction) for marketing purposes can improve customer experiences and engagement and increase brand awareness and recognition (Barrera & Shah, 2023; Dincelli & Yayla, 2022; Dwivedi et al., 2022a; Hennig-Thurau et al., 2022). For instance, Ralph Lauren has created a virtual clothing line within the Zepeto social network and virtual spaces (e.g., an iconic New York City location, Ralph's Coffee Shop, and a live virtual concert) (DeAcetis, 2020). As part of the promotion of Ralph Lauren’s virtual clothing line, customers are able to interact with avatars wearing the 3D clothing collection and taking virtual selfies with them. Such a virtual experience not only allows customers to “try on” products in a virtual store, but also increases brand awareness by reaching new audiences and creating real-time multisensory experiences.

While metaverse has the potential to offer a variety of benefits for businesses, it presents several challenges that organizations need to address. We observe three key unintended consequences triggered by the utilization of metaverse. The examples of metaverse-triggered unintended consequences are summarized in Table 2.

**Table 2:**
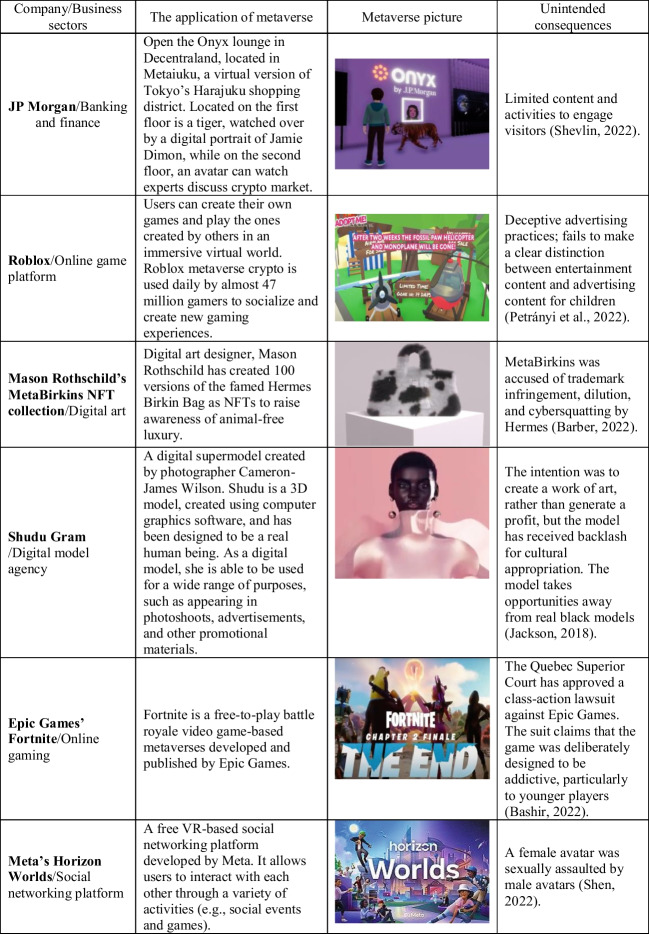
Examples of metaverse-triggered unintended consequences

##### Emergence of Technostress

The value that the metaverse creates is dependent on how users respond to using specialized hardware or software is required to access the metaverse (Hennig-Thurau et al., 2022). Indeed, for social interaction to be more realistic, substantial investment in advanced hardware (e.g., VR headsets and motion controllers) is required for users. It is also essential for users to comprehend the design and functionality of the virtual environment, interact with avatars and purchase products using digital currencies. From the personal use perspective, if users feel nauseous or disoriented while using VR or AR, it can be difficult for them to fully engage with and enjoy the content that is being presented to them within the metaverse environment. This engagement may create technostress, defined as technology-induced stress that an individual experiences (Issa et al., 2022; Salo et al., 2022).

##### Technical Complexity

Creating and implementing metaverse marketing campaigns can be complex technically and require specialized skills and resources to overcome (e.g., 3D digital content creation, blockchain structure and access, and virtualization engines), which may be a challenge for some organizations (Dwivedi et al., 2023a; Ersoy & Gürfidan, 2022). For example, in 2020, Nike launched an AR marketing campaign called “Nike Fit Augmented Reality Footprint”. The campaign used AR technology to allow users to virtually try on different Nike shoes, using their smartphone's camera to visualize how the shoes would look on their feet. To implement this campaign, it required Nike to invest in specialized AR technology, work with a team of developers to create an AR experience and develop a marketing strategy to promote the campaign. However, currently available metaverse technologies may have limitations, such as the need for specialized hardware and high-bandwidth networks, low resolution and interactivity, and difficulty of integration with existing systems, which may adversely affect user experience (Hennig-Thurau et al., 2022).

##### Legal and Ethical Concerns

The use of metaverse technologies may raise legal and ethical concerns such as privacy invasion, data security, and misleading practices. It is possible that metaverse technologies collect data on users’ interactions and behavior, which may raise privacy concerns, data breaches or cyber-attacks if the data is not properly secured or used. In addition, in the metaverse environment, misleading or deceptive marketing contents may be created. For instance, the NGO Truth in Advertising has filed a complaint with the Federal Trade Commission (FTC) regarding Roblox's alleged deceptive advertising practices (Petrányi et al., 2022). The platform lacks a clear distinction between advertising and entertainment content, particularly for children, as well as between sponsored games and individual user-generated content. There are also legal concerns raised about advertiser-backed influencers appearing as avatars in the metaverse, as well as the sponsored nature of virtual events.

**Fig. 3:**
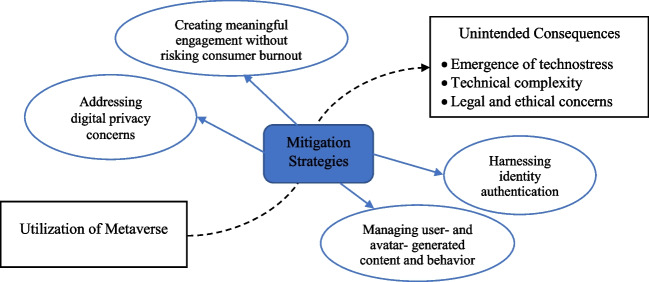
Mitigation strategies of metaverse-triggered unintended consequences

To address these challenges, we uncover four mitigation strategies for addressing unintended consequences of metaverse technologies used in the marketing domain, as depicted in Fig. 3.

#### Mitigation Strategies of Metaverse-triggered Unintended Consequences

##### Addressing Digital Privacy Concerns

There is a growing threat of data breaches and privacy invasions in the metaverse as it continues to develop. It is essential to prioritize user data security when creating new metaverse-based experiences, as failure to do so could lead to widespread consumer mistrust (Krishnamurthy et al., 2022). Metaverse designers must be cognizant of consumer demand for transparency and privacy controls to ensure trust between metaverse consumers and metaverse experiences. For example, a metaverse experience should provide users with the option to opt-in or out of data collection, as well as give them access to view what data is being collected from them. By utilizing consumer feedback to inform their metaverse design, metaverse creators need to work closely with their marketing and IT teams, data analysts and legal professionals to ensure that they have the tools and expertise necessary to use customer feedback to inform their design decisions (Dwivedi et al., 2022a, 2023a). Moreover, there are also concerns about content ownership and privacy in the metaverse, including potential issues with NFT transactions and the legitimacy of cryptocurrencies. To address these issues, it may be necessary to implement technologies such as Confidential Computing and to issue digital smart contracts for certification of authenticity (Krishnamurthy et al., 2022).

##### Creating Meaningful Engagement without Risking Consumer Burnout

The metaverse has the potential to connect businesses, brands, and consumers. Decentralized Autonomous Organizations (DAOs) and NFTs are examples of how metaverse business models can be new types of borderless and global businesses. Taking full advantage of the metaverse's potential will require executives to adjust their decision-making processes to accommodate digital business models (Williams, 2022). As users remain reluctant to use or accept the metaverse (Issa et al., 2022), executives need to provide clear justifications for users to spend time in virtual spaces and set clear engagement goals rather than stressing the metaverse concept per se. Simply inserting an existing business model into the metaverse is not enough to ensure its sustainability in the virtual space. A successful example is that Epic Games’ Fortnite organized a Travis Scott live Fortnite concert in 2020 with over million players attending. This metaverse event is seen by Epic Game as a mere extension of its core game development.

The metaverse allows users to interact and engage with one another and is being used by marketers to create meaningful consumer engagement and drive-up consumer aspirations (Hennig-Thurau et al., 2022). This can be achieved through co-creation of content, “conversational commerce” (Miao et al., 2022) and haptic experiences (Luangrath et al., 2022), product testing, corporate social responsibility activities, and brand mascot virtual interactions. However, it is important for marketers to be mindful of their audience and the amount of time and effort they are asking them to put in. Providing a balance of activities, offering a mix of interactive experiences, allowing consumers to control their own level of involvement can avoid consumer burnout.

##### Harnessing Identity Authentication

Identity authentication is an important issue in the metaverse, as it allows users to establish their online identities and ensures that they are who they claim to be. The metaverse offers a range of authentication opportunities beyond traditional physical identification. With the rise of virtual influencers, digital avatars, and fake IDs, verifying identity within the metaverse can be much more difficult than in the real world. There is a wide selection of biometric and cryptographic solutions that may be implemented in the metaverse to help ensure security and prevent identity theft. For example, users may use facial recognition, voice recognition, eye scan technology, or other biometric authentication methods to prove their identity when accessing certain digital worlds. Additionally, metaverse-specific encryption keys can be used to secure digital objects such as NFTs, ensuring that assets are not stolen or compromised. While metaverse identity theft is still a possibility, these authentication methods help to protect against metaverse identity theft and provide a greater measure of security for human users.

##### Managing User- and Avatar- Generated Content and Behavior

Virtual influencers are digital avatars or characters that are used to represent real people or brands on social media or in virtual reality environments (Miao et al., 2022). To manage the identify and behavior of virtual influencers in metaverse environments, firms need to set clear guidelines and expectations for the use of virtual influencer (Dwivedi et al., 2023a). This should include guidelines for social media posts, interactions with fans and followers, and any other public-facing activities. Firms also need to take action if the virtual influencer’s behavior goes against the guidelines or expectations set. This may involve removing inappropriate content, issuing warnings, taking more severe action if necessary, and monitor the virtual influencer's activities and interactions on a regular basis (Kim, 2021).

In the metaverse, sexually explicit activity is a growing concern. For example, the metaverse has seen a rise in the prevalence of cybersex trafficking, which involves the sale and purchase of virtual sexual services. The victims are vulnerable people from marginalized communities who are coerced into performing online acts that are then broadcast to paying customers. This form of exploitation is particularly concerning as metaverse users can remain anonymous and thus, evade law enforcement. Given the potential for abuse and exploitation, it is important to take steps to protect minors from viewing or engaging in sexually explicit content in metaverse environments (Kim, 2021). Strict regulation of metaverses should be implemented through age verifications systems, regular monitoring of virtual clubs, and the use of artificial intelligence-based technologies to detect and remove inappropriate content. Moreover, metaverse users should be encouraged to report any suspicious activity or behavior they witness in virtual clubs. Such proactive measures will ensure that metaverse environments are safe spaces for users of all ages.

### Scoping the Future of Dark Side of Metaverse – A Thematic Overview– Abhishek Behl, Gareth H. Davies and Vincent Dutot

The metaverse has a long history of evolution, but it has recently gained attention from businesses across all sectors. While the growth in interest in the metaverse has been phenomenal and studies have dissected the metaverse into multiple dimensions, its exploration of the dark side remains less explored. The vices of metaverse can be divided into the following categories.

#### Psychological Issues

The metaverse offers a parallel world to its users, which often keeps them a deluded state about what is real and what is fake. Studies have shown that excess time spent in virtual world impacts users' psychological well-being and often results in anxiety, technostress, etc. (Dwivedi et al., 2022a, 2023a; Oh et al., 2023). It could also lead to social disconnections and relationship issues in the real world. As the metaverse world starts growing with the population of people, it is expected to impact their real-world mental health. Drawing from the gaming world, it is also expected that the meta version of people will face psychological issues in the metaverse world, like bullying; a toxic environment with gender imbalance avatars (Gadekallu et al., 2022; Oh et al., 2023). Thus, these aspects call for exploring psychological issues from the users' perspective in the metaverse and real world.

#### Security and Governance Issues

The growth of gadgets helps people transition into the metaverse world. While Web 2.0 somewhat restricted people from sharing certain forms of data such as contacts, finances, and engagement, the gadget-driven metaverse world adds a layer of data that firms can collect. Avatars-driven metaverses and the virtual world make users travel via technology. The pseudo-space time makes them act in situations that the human body is not accustomed mainly, generating new forms of data that are often unregulated and unmonitored (Wang et al., 2022). Studies in virtual worlds indicate an immediate need to assess the security and governance issues of data, its breach, and its misuse (Dwivedi et al., 2022a, 2023a; Wang et al., 2022).

Moreover, the current technology does not restrict users to experiencing metaverse under a particular age bracket. On occasions where the age is validated, the systems' design is weak in verifying age and user profile (Gadekallu et al., 2022). There is a need to assess security and governance from an organizational and individual perspective.

#### Theoretical Foundations

Recent studies have used some theoretical foundations to explain specific actions of users and organizations in the metaverse; however, the choice of using theories is often not debated. Current metaverse focussed studies deal with a combination of 5 assumptions as proposed by Alvesson and Sandberg (2011): in house assumptions; root metaphor assumptions; paradigm assumptions; ideology assumptions; and field assumptions. Although most studies don’t consider these areas as a risk or challenge, it is important to ensure the literature addresses these areas to develop arguments appropriately and not develop incorrect theoretical paradigms (Kozinets, 2023). Drawing from the lens of problematization (Sandberg & Alvesson, 2011), it is critical to not rely on confusion spotting, neglect spotting, or application spotting.

#### Health Issues

With the growing population on metaverse, keeping the real world interactions at stake, health issues would become the primary concern for users. As the metaverse is experientially new to the users, there lies a risk of higher degree physical catastrophes. The exposure to disrupted emotional simulations could impact their sleep cycles; obesity and cognitive learning skills in the real world (Garavand & Aslani, 2022). This would further trigger personality issues in the real world. Studies have also indicated that people who play excessive games tend to deal with issues like split personality; and mismatched expectations (Gadekallu et al., 2022; Kerdvibulvech, 2022).

#### Ethical Issues

The metaverse, unlike the real world, is not developed with law and order as an integral element of the infrastructure. Entities even target the decentralized version of parallel metaverses to breed issues like cyberbullying; fraud; financial scams; neurological manipulations; and unconventional use of technology (Zallio & Clarkson, 2022; Smaili and Rancourt-Raymond, 2022). Drawing from the tenets of responsible artificial intelligence (RAI) and explainable artificial intelligence (XAI), it is crucial to explore the responsible metaverse (RM) and explainable metaverse (XM). The metaverse needs to be explored from perspectives of morals; responsibilities; trust; behavior; principles; relationships; choices, and reliability. The asymmetrical growth of technology and business expansion also needs to be explored using moral policing.

#### Knowledge Management Issues

Knowledge management, in its contemporary form, focuses on people, processes, content, and strategy. The metaverse will face issues related to developing knowledge libraries and pathways due to its unorganized and unstructured form of data (Wang et al., 2023). The current form of metaverse empowers users to present and project knowledge and information controlled by them rather than a pre-defined system. This promotes information asymmetry, knowledge hiding, and knowledge mismatch, as it would be patchy at the moment (Buhalis et al., 2022). The form of data and knowledge patches would also have concerns regarding their reliability and validity. It would raise difficulties in fraud detection procedures, personal information protection, setting and controlling personal data; maintain real and virtual profiles of users, to name a few.

Having said this, the dark side of the metaverse needs to be studied from a scoping review of linking the following themes:

- How can behavioral and social psychology be used for users inside and outside the metaverse over a long time?
- How do users in the metaverse evolve their psychodynamics and cross-cultural psychology in a fuzzy and unorganized environment?
- How can data breaches and identity theft be understood and controlled in the dark web and misrepresented and delusional presence and activities of users in the metaverse?
- How can new theories be generated for understanding the scope and dimensions of the dark side of the metaverse?
- Will the metaverse lead to new diseases and resurface old ones, and is the healthcare industry ready for it?
- How do firms control the unethical behaviour of employees and users in the metaverse? Can spending time in the metaverse impact their real-world ethics?
- Can knowledge hiding be considered an asset in the metaverse? Does the metaverse change the personality of the being temporarily or permanently?

### Murky Side of Metaverse: Implications and Research Questions—Neeraj Pandey, Mihalis Giannakis and Ramakrishnan Raman

The online world is getting transformed with the advent of metaverse-based platforms (Dwivedi et al., 2022a). The metaverse, despite its virtues, has several dark side dimensions. The cognition and awareness of these negative facets would aid in building appropriate checks and would go a long way in seamlessly managing metaverse-based platforms. There is a need for organizations to align the conceptualization, implementation, and database usage of their metaverse platforms with the legal provisions of the state. Firms must have clarity that the metaverse is a medium to reach out to its stakeholders in an efficient manner and not an end itself. The focus of the service or product should be providing a superior customer experience where the technology is just an enabler (Ramasundaram et al., 2023). The deployment of hardware and software as an integral part of the metaverse environment, at times, takes away the level-playing field that digital marketing brought to startups and medium enterprises against the cash-rich larger conglomerates. There have been several incidences of bullying and assault on the metaverse platforms (Table 3). The regulation of the metaverse by a neutral body is an open question. It would help to adjudicate and regulate any reported incidents of bullying, fraud, and violence by the avatars on the platform. There should be a provision that the things that are unlawful in the real world, like advertisements of guns and prohibited drugs, should also be a punishable offense in the metaverse platforms.

**Table 3:** Dark side of metaverse

Incident	Metaverse platform	Details about the incident	More details
Assault	MetaVenues	A lady was assaulted by male avatars as she entered the metaverse environment. She was verbally and sexually assaulted in the virtual space, which left her traumatized	https://cybernews.com/editorial/in-the-metaverse-the-attack-surface-expands-to-your-brain-interview/
Bullying	Meta VR Chat	It included the use of unparliamentary language, racism, and pornographic content by the users. The visitors on the platform also included minors	https://counterhate.com/blog/new-research-shows-metaverse-is-not-safe-for-kids/ https://twitter.com/CCDHate/status/1491516503937589251
Harassment	Microsoft AltspaceVR	The male member intruded on the ongoing discussion between female members. It made the female member uncomfortable as he entered her avatar’s personal space and chatted inappropriately. Microsoft built the AltspaceVR bubble to restrict people from entering other person’s personal space	https://techcrunch.com/2022/02/16/microsoft-shuts-down-altspacevrs-social-hubs-to-combat-harassment/ https://cybernews.com/editorial/the-dark-side-of-the-metaverse-taking-your-nightmares-online/
Digital addiction and depression	All metaverse platforms	Excessive screen time and AR/VR usage led to digital addiction and depression, besides other mental health issues	https://www.forbes.com/sites/forbesbusinesscouncil/2022/08/17/the-metaverse-and-mental-health-supporting-employees-in-virtual-environments/?sh=67707d9ece4e https://www.familyaddictionspecialist.com/blog/a-new-age-of-digital-addiction-what-the-metaverse-means-for-mental-health-and-digital-addiction
Hacking and Blackmail	Roblox	Hackers infected Roblox and blackmailed the site owners into paying them in its virtual currency Robux. Hackers also stole Robux and gears from individual accounts and tried to sell them on the Discord channel for globally tradable bitcoins	https://www.vice.com/en/article/88gd4a/roblox-beaming-hackers https://techmonitor.ai/technology/cybersecurity/roblox-hack-data-leak

#### Industry-Specific Dimensions

The metaverse has brought a paradigm shift in customer, peer, and organization-level interactions. It is a three-dimensional immersive online platform as compared to two-dimensional interactive social media channels like Facebook, LinkedIn, and Instagram. The metaverse environment is akin to the real-world physical space leading to superior customer experience and efficient interfaces. It facilitates hyper-customization and fun in online interactions by providing options to choose one’s own avatar. The increased use of the metaverse by individuals, organizations, and government bodies is leading to an urgent need for addressing and checking the dark side of this online phenomenon. The industry-specific dark-side issues and challenges are enumerated below:

##### Gaming and Metaverse

Gaming was the earliest industry where the metaverse type of mechanics was implemented. Roblox, Zepeto, and Fortnite were such popular gaming sites. The gaming sites are prone to cyberattacks to steal the user profile information besides taking away their virtual gear and earning from games like Robux, ZEMs, coins, V-Bucks, etc. (Table 3). The excessive use of metaverse platforms has led to mental health cases, particularly among children and young adults (Dwivedi et al., 2022a). Natural movement, including exercises and physical sports, has reduced due to the extreme indulgence of metaverse-based gaming platforms. Addiction to such platforms may also lead to attention deficit and eating disorders among heavy users. The excessive use of headphones and AR-VR headgear on these new-age platforms has led to an adverse neural impact on heavy users (Wells, 2021).

##### Digital Marketing and Metaverse

The initial experience of firms regarding digital marketing on metaverse platforms like Decentraland and Roblox has been mixed. Many organizations had confusion over applicable attribution models and returns on investment (RoI) in terms of sales conversions. The issue of bots and impersonation on metaverse platforms, besides the growing digital divide, was another issue that needs to be addressed. The increased use of the metaverse for digital marketing, has led to increased energy consumption and thus an increased carbon footprint. Privacy is another major concern on metaverse platforms. The granularity and amount of data collected in the three-dimensional metaverse environment is much more than the traditional two-dimensional social media platforms. The tracking using digital marketing tools to steal metaverse users’ data is a real threat. Privacy in social networking platforms is a subjective phenomenon, and perception may vary as per the individual (Pandey & Gudipudi, 2019). However, metaverse platforms have to devise certain rule and protocols for ensuring users’ privacy to provide users sufficient confidence in metaverse platforms and allow them to interact freely. The metaverse has also led to increased use of NFTs and cryptocurrencies that have given rise to price speculation and increased financial risk to buyers about their future valuation (Wilson et al., 2022).

##### Healthcare and Metaverse

Besides advertising, entertainment, education and healthcare are other domains where the use of metaverse platforms is expected to grow in the coming years significantly. Lack of human touch and empathy, besides privacy concerns, are leading to adoption challenges of metaverse-based healthcare delivery systems. The legal compliance and safety issues in transferring patient medical records to other departments and hospitals in a metaverse environment are yet to be navigated (Chengoden et al., 2022). Metaverse-based healthcare databases would be prone to cyberattacks which may lead to the loss of critical patient medical records and financial details, which in turn can impact the treatment and insurance claim process. There is also reputational risk for the healthcare service provider and regulator in such violations.

#### Implications for Practice

The metaverse, as an idea and its implementation, is progressing to maturity. The negative aspects are part and parcel of this digital journey. Metaverse users would expect the regulations and rules of the environment be established. There is a need for the conceptualization, development, and validation of the cybersecurity index. This index will not only authenticate the metaverse platforms but will also reflect its degree of validity and safety. The incidents of bullying and fraud are increasing as more and more new users are joining metaverse platforms. The ecosystem requires behavioral measures to be taken by metaverse organizations besides functional measures such as code-based entry permissions for authentication. These initiatives would help to minimize toxic behavior by miscreants on metaverse platforms. There is also a necessity to sensitize corporate entities, particularly startups and medium-sized firms, about the dark side of the metaverse to ensure they are fully aware of the applicable rules, regulations and laws.

Based on the above discussions, the following research questions (RQs) must be explored by future researchers working in this domain:

- RQ1: What should be the steps in the development of a cybersecurity index for measuring the authenticity of a metaverse platform?
- RQ2: What should be the strategy of the corporate world to sensitize the masses against falling to fraud on metaverse?
- RQ3: What are functional and behavioral measures to check bullying in a metaverse environment?
- RQ4: What are changes required by metaverse marketplace platform developers to enhance user well-being?

### The darkside of the metaverse today—Leighton Evans

The Metaverse refers to a future of digital media dominated by VR and AR that will involve a significant shift from activities in the physical world to virtual worlds. The eventual metaverse, if it ever exists, will be a persistent virtual world users can enter and exit at will and include a wide variety of activities, ranging from commerce to theatre to exercise to gaming. Each domain that the metaverse appropriates has already a myriad of issues and problems, and these are likely to be replicated and potentially amplified in the metaverse. When considering the dark side of the metaverse, governance entities need to begin grappling with the consequences of what will happen if even a fraction of the metaverse vision comes to pass. The metaverse is simultaneously a return to an old-fashioned understanding of digital media and an ambitious futuristic vision which brings several new issues to the table. The metaverse is also an object that in many forms already exists. Much of the groundwork has already been established to understand what a metaverse might mean for many parts of our lives (Evans et al., 2022). There are numerous disconnected digital spaces in which people can engage in isolated practices, which the metaverse will look to join up into a unitary experience.

The metaverse will need to be a space for social interaction. It is hardly surprising that the company behind the world’s largest social network Facebook—Meta is leading the charge to the metaverse. Since the emergence of Friendster more than 20 years ago, social networks have been transplanting many everyday social functions and activities from ‘meatspace’ to a digital space for interaction. The creation of an experiential and virtual world that can be embodied by the user is a logical next step in the social media experience. As is only too well known, existing social media environments bring a host of issues to the metaverse experience: the exploitation of users in the name of surveillance capitalism (Zuboff, 2018); the death of privacy (Fuchs, 2015); the manipulation of voters in 2016 (Cadwalladr & Graham-Harrison, 2018); addiction (Hou et al., 2019); a nihilistic attitude to the world (Gertz, 2018); boredom in the name of neoliberalism (Kingwell, 2019); an interface designed to make us sad (Lovink, 2019) and many other issues far too numerous to list further. Early steps to transplanting social interaction in metaverse-like environments have raised new issues. In VR, applications like VRChat equally provide an indication of how worlds like Meta’s Horizons (the company’s much hyped social VR platform) might be experienced. At the same time, these worlds also tell us something important about how metaverses may be experienced*.* The kinds of behaviour commonly seen in VRChat and other worlds (characterized by low-level trolling to serious infringements on personal space in VR and sexual harassment), raise serious questions for the social experience of the metaverse if the issues seen in other social worlds become endemic to an all-encompassing digital world. Crawford and Smith (2022) report that VRChat’s lax rules has allowed children into ‘virtual strip clubs’. As early as 2016, Jordan Belamire reported being groped in VR when using the social VR gaming application QuiVr on the HTC Vive (Belamire, 2016). Recently, Basu (2021) detailed how the avatar of a female beta tester was groped on Meta’s Horizons platform. The induced immersion, presence and embodiment can make the experience much more painful for the victims of malicious behaviour. While avatar bodies cannot physically hurt another avatar, the perceived physicality may carry a greater threat to a user than a tweet or comment (Bailenson, 2018, p. 202).

Much like social media, digital games have a long and rich history of sustained presence as both cultural artefacts and for players of games. The notion of shared, continuous gaming experiences with other players has become an important aspect of contemporary gaming. Accordingly, the online game industry is a lucrative business, and customer loyalty over time is critical. Understanding how games maintain players’ presence is therefore important as a precursor to understanding how Meta may do this with the metaverse. Games and gaming will be critical in establishing what people will do in the metaverse. It is in this light that gaming worlds may become fascinating indicators of what kind of place the metaverse would be for all. From console games online to MMORPGS*,* there are significant questions about the metaverse that are raised by a critical evaluation of gaming. David Sudnow (1983), when describing videogames, noted that games depict microworlds where an augmented version of the player has bodily interaction with the game. A game player engages; in an embodied manner; a game player is part of the game. This is exactly what is desired from the metaverse – engagement distributed across the body. The many digital worlds are incredibly close to the form that the metaverse will take embodied, active spaces where augmented individuals interact fully with a digital environment. The mission of the metaverse is much like McKenzie Wark’s (2009) argument that the ideological consequence of games is to transform reality into a totalizing game space. While games clearly point to several things that the metaverse should and needs to do, there are also aspects of digital games which are going to be deeply problematic. The notion of playbour (Goggin, 2011; Kücklich, 2005) – playing for free or paying to play and that labour being leveraged for profit – will likely be embedded in the metaverse. The gaming industry is often characterised as a toxic, hyper-masculine culture. Anthropy (2012, p. 7) argued that game publishers perpetuate a dangerous cycle by repeatedly creating games for young men already entrenched in the existing culture of games. This culture would not be conducive to inclusivity in the metaverse. For Humphreys (2019 , p. 837), the gaming industry is diminished by misogyny, making it risk adverse and marginal which is not where Meta needs to be as a company. Meta will also need to challenge some dominant ideas and ideological constructions in games. According to Chess (2017) the idea of ‘player one’ is a male, white, affluent, able-bodied, and heterosexual consumer of commercial releases. Player two is a woman that enjoys mobile games. This would not be a tenable position for the metaverse.

The exploitation of users is a common theme which may characterise the metaverse. The metaverse could simultaneously offer users freedom to build their own parts of a virtual world while also exploiting their labour. The impression given by promotional materials is that we shall be presented with a fully formed, fully realized world. In other words, the metaverse will provide a vast and expansive playground for users to explore. The history and business models of the protagonists in this space, however, suggests otherwise. The content of social media has been us; user generated content (UGC) both of and by ourselves, which is then sold back to us in a recursive feedback loop of commodification and commercialization. In the metaverse, we can expect more of the same – building our own materials much as we do in social media, to be used for likes and comments from others that may take a different expressive form but will be for the same financial gain. Yet, there are also nuanced differences in this virtual environment that warrant circumspection. As demonstrated by Zuckerberg’s presentation metaverse in October 2021, that detailed Meta’s VR social platform Horizon, these worlds are not simply built by the communications of users, but more importantly the building of these spaces are physically performed through virtual tools that users can employ to construct and develop these interlinked virtual spaces. Digital environments already exist which illustrate the issues that will arise from UGC being a fundamental part of the metaverse. In particular, the notion of the exploitation of unpaid labour is critically important here. In particular, there have been several examples of copyright infringing UGC which has contributed to exploitative child labour practices and the creation of unsafe digital environments on Roblox (Parkin, 2022). A metaverse might accelerate this on a vast scale.

Being in a metaverse will have to afford the possibility of people meeting one another, dating, forming a relationship and maintaining that relationship. The impact of applications and social media features on dating are already well documented. One may fear the ‘Tinder-isation’ of dating, but the metaverse may make this the de-facto method of choosing and meeting a partner. As Bergström (2021) argues at length, the mediation of dating via applications has led to a privatisation of intimacy. The metaverse will need functionality for embodied avatars to experience intimacy. Whether this is implied or actual will be a function of the equipment available for the user. One can already envision a multi-tiered hierarchy of intimacy, where wealthy users can afford sophisticated haptic systems designed for physical intimacy while basic users have little but their words and the non-verbal communications of their avatar to express their emotions. Less affluent users would be explicitly limited in their ability to communicate with others, and in doing so, experience lives that feel more meaningful. In a world where the perception of the platform and advertising concerns will be of paramount importance and arousal is intensified by the use of VR in particular (Evans, 2022), it is possible that some visions of the metaverse will bring with them a new puritanism around sex, sexual identity, and the expression of sexual behaviour between users.

### Children, Young People and the Metaverse—Nina Jane Patel

Technology is a part of the everyday lives of children and young people (CYP), influenced by parents, educators and many other social interactions (Tootell et al., 2017). As the next evolution of technology, the Metaverse, develops, the early-stage activations are attractive and seemingly designed to engage with younger generations such as Z and/or Alpha (Dalot, 2018).

Indeed, technology firms are jumping on the metaverse trend—Nvidia Omniverse, Meta Horizon, Fortnite (Epic Games) and Roblox to name a few (Kim, 2021) highlighting opportunities and efforts to gain increased engagement with younger generations. Brand activations are often aimed to engage in fun, entertaining ways; Oreo introduced “Oreoverse” an Oreo-themed world of games and cookies on Meta Horizons (Baar, 2023), Nike built ‘Airtopia’, a kids’ world in the metaverse, recently, children fashion brand Balabala announced its entry into the metaverse, creating a hyper-realistic digital brand ambassador named 'Rainy’, other kids’ brands, including Hasbro and Mattel, have also announced their Metaverse strategies (Shaikh, 2022) as have Lego and Disney (Solis & Danise, 2022). It’s all fun and games, but there are huge trust issues when it comes to technology companies and children (Restuccia & Tracy, 2023). England’s Commissioner for Children made a series of worrying discoveries during the course of their research, making clear the urgent need to protect children from harms online (Children's Commissioner for England, 2022). Not only did the commissioner find that it is “quite likely” for eight year olds to come across pornography online, but also learned of the “insidious” violent content that CYP are confronted within current digital environments (Turner et al., 2022).

The UN Convention on the Rights of the Child (UN Convention on the Rights of the Child - UNICEF UK, 1992) has 54 articles that cover all aspects of a child’s life and set out the civil, political, economic, social and cultural rights that all children everywhere are entitled to;

- *the right to relax and play (Article 31)*
- *the right to freedom of expression (Article 13)*
- *the right to be safe from violence (Article 19)*

In the context of the Metaverse and its vision of convergence of the physical and digital (Li et al., 2022) and its cumulative impact on CYP’s futures; their relationships, education, careers, hopes, dreams, aspirations and how they see their life unfolding – considerate methods of the Metaverse and its integration into the lives of CYP is vital and urgent.

#### The Illusion of Falsity

The Metaverse is a perpetual and persistent multi-user environment that combines physical reality and digital virtuality. It is based on the convergence of technologies, such as Virtual Reality (VR), Mixed Reality (MR), and Augmented Reality (AR), Digital Twin, and Blockchain, that enable multisensory interactions with digital objects, virtual environments, and people (Mourtzis et al., 2022). The components of the Metaverse, particularly virtual reality (VR) progressively extend CYP’s view of physical reality, for instance by altering their identity and digital appearance via avatars; by immersing them into synthetic environments to engage with other humans; or, in the future, by generating a sense of touch within their brain when they interact with a digital object (Hilken et al., 2017). These new technological components that comprise the metaverse offer an opportunity for CYP to engage in networked environments that can accommodate compelling synthetic virtual engagements offering viscerally heightened experiences, authentic content and social meaning (Park & Kim, 2022).

For example, evidence suggests that VR experiences can lead to positive social and psychological outcomes (Markowitz & Bailenson, 2021) and deepening learning of societal issues (Markowitz et al., 2018) and building empathy (Herrera et al., 2018). Furthermore, research shows that VR provides high fidelity sensory information and that the brain often treats a virtual experience in a similar manner to a real experience (Blascovich et al., 2002).

Technology developers have identified that success will depend on the acceptance that the virtual and physical are sensorily similar and that the metaverse thus represents a genuine reality in which users can socialise, work, and play (Golf-Papez et al., 2022). In other words, to be compelling, the metaverse will need to suspend the disbelief and abandon the notion that synthetic experiences are inherently “false” to prevent discounting the value of a technology-enhanced reality (Hilken et al., 2017). While digital experiences can be psychologically real to the person immersed in the metaverse (Wolfendale, 2007), it is often discounted by outside observers because no activity takes place in the physical world. Such discounting stems from traditional views of falsity, which assume that only physical experiences (i.e., those derived using unaided biological senses like sight, hearing, touch, taste, and smell) are real (Ross & Ward, 1996) and that synthetic, digital forms of experience are imaginary, inconsequential and are not real.

Problems lie ahead if we underestimate the psychological impact of the convergence of physical and virtual worlds while the lines between are increasingly blurred as fidelity improves (Heller, 2020). For CYP, the problem with the traditional perspective of falsity is that it denies the authentic sense stimulation created by immersion, presence and embodiment (Park & Kim, 2022) in the multi-sensory synthetic metaverse. CYP who adopt an alternate identity using an avatar (e.g., to become a superhero-like character instead of their usual physical self) likely integrate the qualities of this avatar into their persona and use those qualities to achieve goals and objectives in the metaverse otherwise not possible in physical reality. The potential attachment to metaverse experiences and interactions as it can be felt as very real and it holds value to CYP as it is expressive of self-identity and self-conception and should therefore be accorded the moral significance we give to real-life attachments that play a similar role (Wolfendale, 2007).

I put forth that to naively underestimate this alternative persona for the child or young person along with its related goals and objectives of human interaction, connection and communication as false, fails to appreciate the implications on psychological and developmental areas of concern, wherein digital relationships acquire meaning from child's perspective. Therefore, the traditional view of falsity of the digital realm is inherently problematic.

#### Empowering CYP in the Metaverse

A more nuanced perspective on falsity, for stakeholders including educators, parents/caregivers and regulators, is one that acknowledges that metaverse experiences will be felt, fully immersed present and embodied and that this immersion will deliver an experience that is equal in value to the physical experience.

The continuation of the illusion of falsity will support the continued disassociation and online harms and deviance experienced by CYP in the current state of the internet.

Alarmingly the CC-Driver 2021 report, which is one of the largest studies to date exploring youth cyber criminality and is informed by 5 key disciplines: cyberpsychology, criminology, psychology, neuroscience, and digital anthropology, demonstrates and confirms what is widely known, that young people are immersed in technology and our approach to supporting CYP with their relationship to and within technology needs to shift e.g. 47.76% of CYP report to have engaged in a behaviour that could be considered criminal and offensive when online (Davidson, et al., 2022). Research shows that adolescents participate in cyberdeviance, risky or harmful behaviours and cyber crime—1 in 2 respondents (children) watch pornography online, 1 in 5 participate in sexting, 1 in 7 have self-generated sexual images and are participating in cybercrimes such as online harassment, cyberbullying, revenge porn, identify theft and racist/xenophobic speech (Davidson, et al., 2022).

Furthermore, the anatomical, physiological, and developmental changes which arise as children mature through childhood and adolescence support the need to develop new technologies that meet the specific requirements of CYP. Failing to involve CYP during the development of technology increases the risk that the outcome falls short of their expectations and needs, leading to rejection of novel interventions (Wheeler et al., 2022). The traditional view of falsity of digital interactions fails to recognize its potential to empower CYP and to unlock unique opportunities for value creation in the metaverse while building bridges between the physical and digital worlds to support CYP to create healthy relationships with and within technology.

#### Summary

The metaverse is upon us. It is presented as the biggest opportunity for modern business since the creation of the internet (Charlton, 2022). Soon it will be as omnipresent, for CYP, as TikTok, Instagram, and Meta (Hirsh-Pasek et al., 2022). CYP will form friendships, develop relationships, play, learn, explore, be creative and essentially grow up with the Metaverse. Childhood has long been identified as a key transitional developmental period and it is imperative to develop an understanding of how the medium of the Metaverse impacts CYP's psychological and physiological development.

It is essential that future research and resulting products that are delivered into the hands of CYP focus on better understanding the benefits and limitations of Metaverse technology components to ensure that the technology is fit for purpose and does more good than harm.

“A young child, of 8 years old, asks her mother “Is the metaverse real?”

To answer this, I suggest that we reconsider the acceptance of digital falsity. It is vital that we map out a future where we develop responsible strategies to support the long term development and psychological well being of CYP as they are raised with the metaverse.

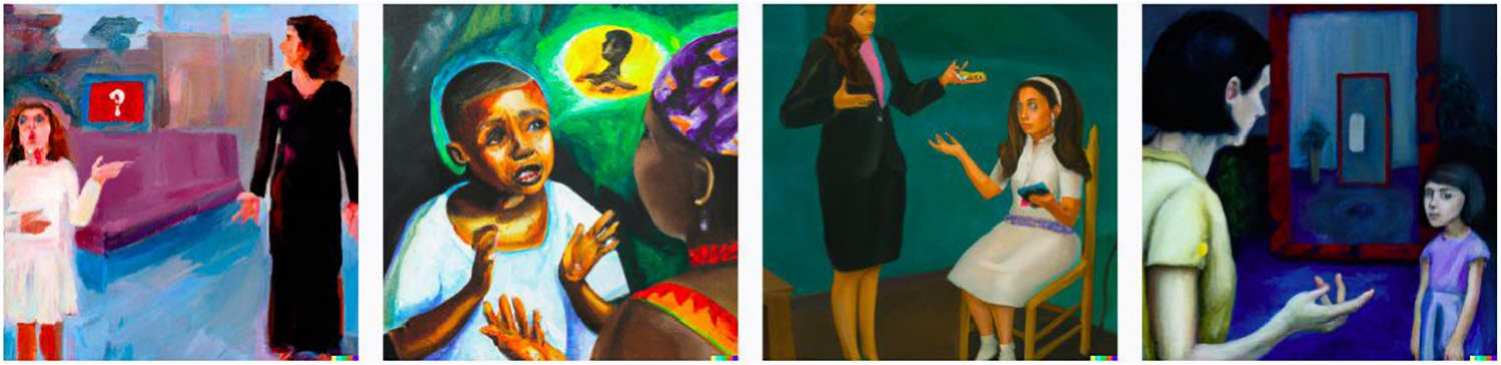


DALL·E—AI system that creates realistic images and art from a description in natural language—when prompted with *“an oil painting of a young child, of 8 years old, asking her mother “is the metaverse real?" “*

## Discussion and Implications

### Synthesis and Identification of Common Themes

In this section, we first identify main themes that emerge from the contributions in this work.

#### Multiple Physical, Mental and Social Consequences of the Metaverse

The metaverse can give rise to a number of adverse physical, mental and social consequences. Such effects have been highlighted in Sectsions 2.3.2, 2.1.1, 2.9.1 and 2.14. Drawing on lessons from other technologies, Section 2.9.1 has pointed out the possibility that technologies used in the metaverse can lead to adverse health and emotional consequences. Section 2.1.1 discusses several adverse effects of XR devices on human health, such as vision problems (Rauschnabel et al., 2022b) as well as increased stress and psychological and physiological discomforts. It is argued in Section 2.3.2 that if the metaverse involves replacing physical activities by virtual activities, metaverse users may experience adverse physical and mental health outcomes. Section 2.3.2 highlighted that increased metaverse adoption will likely lead to a decrease in social interactions.

#### More Adverse Effects on Children and Adolescents

More adverse effects on children and adolescents has been a common theme of various contributions in this paper. As discussed in Section 2.13, online virtual world platform VRChat has allegedly allowed children to enter into ‘virtual strip clubs’ due to the lax rules of the platform. Section 2.1.1 and 2.1.14 noted that children are more likely to be addicted to the metaverse. As Sections 2.5.1.6, 2.8.1, 2.9.1 and 2.11.5 make clear, cyberbullying (also referred to as metabullying in Section 2.1.3) and harassment are likely to be serious adverse effects that children and adolescents may face in the metaverse. Furthermore, as metaverse immersive technologies engage different senses, the effects on victims are likely to be amplified.

#### Security and Privacy Issues

Another common theme is that metaverse users are likely to face security and privacy challenges that are different from the non-metaverse Internet. Section 2.10.2.1 emphasizes the importance of addressing security and privacy concerns in the metaverse in order to gain consumer trust.

Due to some unique aspects of the metaverse, Section 2.12.2 has called for the development of a cybersecurity index for measuring a metaverse platform’s authenticity. As reported in 2.12.1.2, some metaverse platforms such as Roblox have faced cyberattacks in which criminals have stolen the virtual currency Robux from individual accounts and attempted to launder this via other more mainstream cryptocurrencies. Some of the security challenges can be attributed to design issues. As explained in Section 2.5, the operational technology used in the metaverse lacks any design guidelines for cybersecurity and privacy.

It is also important to note here that large language models (LLMs) or generative artificial intelligence (GAI) tools such as ChatGPT (for further details about ChatGPT refer to Dwivedi et al., 2023c) have reduced the entry barriers for cybercriminals, which is further likely to increase cyber-threats facing the metaverse. Cybercriminals are reported to be using ChatGPT to create new Trojans. According to cybersecurity researchers, many cybercriminals with no software development or programming skills had been using OpenAI’s technologies for malicious purposes (Kshetri, 2023a, b).

Sections 2.1.3 and 2.2. note that the metaverse makes it possible for marketers to track the movements of eye, pupil and other body parts (Techtarget, 2022) as well as the emotional aspects of consumer behavior, which provide more sensitive personal information and hence more severe consequences of privacy violations. Section 2.4.2 provides further insights into this phenomenon by focusing on the roles of AI algorithms in enabling the measurement of indicators that are currently untrackable such as the movements of legs (Winkler et al., 2022).

#### Regulatory and Governance Gaps

As emphasized by prior research, regulations related to privacy, security and other aspects of the metaverse need to be updated to minimize various risks (Hughes et al., 2023; Polyviou & Pappas, 2022). Consistent with this idea, several contributions (e.g., Sections 2.1.2, 2.4, 2.7, 2.9, 2.9.1 and 2.11.2) have identified regulatory and governance gaps in the current metaverse. As Sections 2.7 and 2.9 explain, such gaps make it hard to monitor and regulate offensive behaviors in the metaverse. Section 2.11.2 describes how the existing regulatory gaps make it possible for marketers to collect new types of data generated by the human body that are not regulated and monitored.

In order to bridge this gap, Section 2.1.2 emphasizes the importance of well-defined regulations to govern the metaverse. As Section 2.9.1 notes, such regulations are especially necessary to protect vulnerable groups such as children. Section 2.4 discusses the downsides of early (over) regulations and argues that investments in research and education might be more effective approaches.

### Research Propositions

In this section, we analyze the key darkverse trends discussed in this article and develop some propositions.

#### Consumers’ Increased Vulnerability to Manipulation and Exploitation

It is noted in Sections 2.8.2 and 2.13 that consumers in the metaverse face higher threats of being manipulated and exploited than the non-metaverse Internet. Section 2.13 specifically warns about more adverse impacts of surveillance capitalism in the metaverse. AI in the metaverse makes it easier to access user-related information, which will increase consumers’ vulnerability to manipulation by organizations (Henz, 2022). Mathematician, logician and cryptographer, Alan Turing stated that a human-level AI’s ultimate test would be to successfully fool consumers into believing that the AI is human. It is also argued that the goal of VR and AR technologies in the metaverse is to fool the senses by making computer-generated content seem like real-world experiences. As explained in Section 2.9.1, XR technologies make users believe that what they are experiencing is psychologically genuine. The above discussion suggests the following:

- P1. *The metaverse will increase consumers’ vulnerability to manipulation and exploitation by legitimate organizations and illegitimate bad actors.*

#### More Negative Effects of Offensive Behaviors in the Metaverse

Sections 2.5.1.6, 2.8.1, 2.9.1 and 2.13 above highlighted the increasing prevalence of offensive acts such as cyberbullying and harassment in the metaverse (Henz, 2022; Qasem et al., 2022; Wiederhold, 2022a, b). While these offenses also take place in the non-metaverse Internet, they have been found to result in more negative effects on the victims in the metaverse. Metaverse technologies such as VR, engage senses such as sight, sound, smell, and touch. This means that the victims of offensive acts such as cyberbullying and harassment are likely to feel that they are truly there (Blackwell et al., 2019). Due to the immersive nature of metaverse technologies such as VR, the effects of such offenses are more comparable to real-world incidents than similar offenses that take place in the non-metaverse Internet (Kothgassner et al., 2017; Wiederhold, 2022a, b). The above discussion leads to the following proposition:

- P2.* Offenses such as cyberbullying and sexual harassment are likely to have more negative effects on the metaverse compared to a non-metaverse environment.*

#### Policy Makers’ and Law Enforcement Agencies’ Lack of Preparedness

The issues of jurisdiction and territoriality to fight online crime and fraud has not been resolved even for the current version of the internet (Cross, 2019). There are also issues related to the conflicts of laws (Clifford Chance, 2022).

These challenges are even more pronounced for the metaverse due to higher degree of interaction and user immersion (Clifford Chance, 2022). To take an example, a young woman in India filed a complaint against the violator of her metaverse avatar. However, it became clear that Indian or international laws cannot prosecute the perpetrator of sexual crimes’ when such crimes involve metaverse avatars. India’s Information Technology (IT) laws, which can prosecute ‘obscene content’ online, is the closest legislation to tackle such crimes. However, compared to the punishment for sexual assault, the perpetrator is likely to receive a much lower punishment under IT laws, which were enacted to deal with the current non-metaverse Internet (The European Institute for International Law and International Relations, 2022).

As noted above, the metaverse also differs from Web2 based social media in terms of the nature of digital footprint. For instance, anonymization techniques can make it more difficult to link data to a particular user and obtain digital footprints (Kim, 2022).

As discussed in 2.6.7, there is the demand for advanced crime fighting technologies and highly qualified and specialized law enforcement manpower for the metaverse. Most law enforcement agencies lag behind in the use of advanced technologies in fighting crimes. Such technologies are even more important in tackling crimes in the metaverse. The rapid growth in non-metaverse online crimes has forced police forces to use most policing resources to address these crimes. These crimes have left police forces with little or no resources to police the rapidly growing area of metaverse crimes (Coker, 2022). It is proposed:

- P3. *Policy makers and law enforcement agencies are currently underprepared to control dark side activities in the metaverse.*

#### Metaverse and the Digital Divide

As noted in Section 2.10.1.2 specialized hardware and high-bandwidth networks are required to take advantage of many metaverse applications (Hennig-Thurau et al., 2022). Many consumers that are unable to afford the required hardware products and lack access to high-bandwidth networks are being excluded from participating in popular metaverse applications such as gaming (Kshetri, 2022b). In June 2022, web3 entertainment company Gala Games announced that its blockchain linked game titles would be distributed through the U.S. video game and software developer and publisher Epic Games Store (Cameron, 2022). Since Epic Games does not host servers in Africa, which puts African gamers at a disadvantage. The servers are far away in other continents. African gamers’ reachability, also known as “ping”, is thus adversely affected, which reduces their game performance. Ping is measured in milliseconds (ms). African gamers playing on European servers are reported to have 160 ms- 200 ms ping, compared to European players’ less than 20 ms ping. A European player can see the African player first and can fight more quickly (Matwadia, 2020).

Likewise, many large companies in diverse sectors are already benefiting from the industrial metaverse (Kshetri & Dwivedi, 2023). However, as Section 2.3.1, explains, companies need to spend hundreds of thousands of dollars to purchase the equipment needed to create a 3D model. Thus, the industrial metaverse is currently beyond the reach of small and medium-sized enterprises (SMEs). The preceding discussion can be summarized as:

- P4. *The digital divide is likely to be deeper in the metaverse compared to the non- metaverse environment*.

### This Paper's Contribution to the Literature

Overall, our work contributes to the growing body of literature on the metaverse by explicating the nature of its dark side. A contribution of the paper is to highlight a number of physical, mental and social consequences such as XR devices’ potential negative impacts via a number of mechanisms (e.g., vision problems, increased stress and psychological and physiological discomforts). Our analysis also reveals while some such effects may also take place in the non-metaverse Internet, the effects are likely to be more severe in the metaverse.

The paper also contributes to the literature on the metaverse by explicitly focusing on the adverse effects on children and adolescents. These effects are illustrated with some novel offenses such as ‘virtual strip clubs’ and metabullying. We also contribute to the research on the metaverse by examining major security and privacy issues, by focusing on the operational and underlying technology.

A further contribution of this work is to demonstrate that there are significant regulatory and governance gaps in the current depiction of the metaverse. A related point is that this paper provides important insights into policy makers’ and law enforcement agencies’ lack of preparedness to deal with and respond to criminal behaviors in the metaverse.

Finally, this paper has contributed to the digital divide literature by by documenting how some key features of the metaverse may worsen the digital divide. For instance, our work provides important insights into expensive devices and resources intensive applications that are required to use the metaverse, which may make it disenfranchise low income individuals and SMEs.

### Implications for Policy

The Darkverse has important policy implications. On the policy front, it is encouraging that several countries have started developing national metaverse strategies realizing the potential of the metaverse to contribute to their national economies (Kshetri, 2023a). However, a close look at their national blueprints to utilize the metaverse indicates that they have mainly focused on the positive side of the metaverse. They contain little detail of the implications of the metaverse’s dark side for economies and societies and little explicit statements to handle such activities. While the Chinese government was reported to be studying a registration system for the metaverse, the main goal has been to prevent metaverse users from influencing wider public opinion rather than controlling the dark side of the metaverse in general (Baptista, 2022; Kshetri, 2023a).

In light of the various challenges posed by the darkverse discussed in this paper, nations’ lack of preparedness to tackle such challenges is a key concern. In a 2022 report, the EU's law enforcement agency, the European Union Agency for Law Enforcement Cooperation (Europol) documented the metaverse’s key policing and legislation challenges (Gibbons, 2022). In the current non-metaverse Internet, policymakers and law enforcement agencies have played catch-up to keep up with the latest developments in technologies and tools (Kshetri, 2021). The Europol has warned against repeating the mistakes in tackling scams, frauds and crimes in the metaverse (Gibbons, 2022). It is thus important for nations to enact legislation to govern the metaverse. Nations should also devote more resources in education and training programs to build law enforcement capacity.

Policymakers also face jurisdictional challenges to tackle dark side activities in the metaverse. Such challenges stem from the anonymity of users and data encryption in the metaverse (Dentons, 2022). Such challenges are even more concerning in a decentralized metaverse that involves blockchain due to the lack of central governance (Kshetri, 2022a). When perpetrators engage in harmful actions in the metaverse, it is often not clear which laws apply and which court in which jurisdiction is the appropriate arbiter (Dentons, 2022). Thus, just like the current non-metaverse Internet (Kshetri, 2021), international coordination is critical for fighting the dark side of the metaverse.

### Implications for Practice

There are also important implications for practice. Many big metaverse platforms have failed to attract users, leading to significant financial losses. For instance, Meta Platform’s business and research unit focusing on VR and AR was reported to lose about $14 billion in 2022, which is expected to lose additional $15 billion in 2023. Consequently, Meta’s emphasis has been recently shifted from the metaverse to AI (Hays, 2023). One possible explanation for the observed underperformance of these platforms is that potentially harmful effects may have outweighed the benefits for users. It is thus important for technology platforms and companies operating in the metaverse to take measures to address potential negative impacts.

Businesses are already facing more demanding customers that place a high importance on measures to protect privacy of their data and ensure cybersecurity. For instance, as early as in 2017, it was reported that about a quarter of medium-sized businesses in the UK were asked by a current or prospective customer about their security and privacy measures (Co, 2017). As discussed in Section 2.10.2.1,*** s***ince the metaverse is likely to evolve as a powerful sales channel, it is important for companies to take measures to enhance security and protect privacy of consumer data in the metaverse and address other dark side issues of this evolving online phenomenon.

Companies operating in the metaverse have a special responsibility to protect children from various threats and reduce the adverse emotional and psychological impact of the metaverse on them. Among major threats children face in the metaverse include cyberbullying, sexual harassment, a lack of privacy and exposure to graphic sexual content, (Deer, 2022). Bad actors were also reported to groom children in the metaverse and persuade them to repeat racist slurs (Tong, 2022**).** Studies have also found that children are mixed with adults in several metaverse spaces (Tong, 2022**).** As mentioned in Section 2.10.2.3, verifying identity in the metaverse is likely to be more difficult than in the real world or the non-metaverse online world. Companies currently lack tools to identify who children are interacting with in the metaverse (Tong, 2022). At the industry level, initiatives could be taken by metaverse companies to develop appropriate metaverse identity solutions to identify children so that they can be better protected from various types of abuses in the metaverse and ensure that their mental and physical health is not adversely affected by excessive engagement in the metaverse.

Finally, companies should take measures to address the digital divide issues in the metaverse. As discussed in Section 2.10.1.2, there are various limitations of currently available metaverse technologies such as the need for specialized hardware and high-bandwidth networks such as 5G. R&D initiatives should aim at developing solutions that can make metaverse offerings more accessible to those that lack access to advanced technologies and infrastructures.

## Concluding Thoughts

Despite a number of economic and social benefits of the metaverse, as discussed in this article, this innovation also has a dark side. There exists the need to be aware of and understand the dark side of the metaverse so that this new innovation can be used to improve how we work, live, learn and socialize. In the end, the wellbeing of consumers should be the highest priority and, at present, the metaverse is not fully serving this purpose. Since the metaverse is evolving, the nature of threats and challenges it pose to individuals, organizations, nations and societies are likely to change over time. Appropriate measures at different levels can help minimize the adverse psychological, physiological and social consequences of the metaverse. Development of national technological and personnel capabilities, enactment of new laws that specifically govern the metaverse, a higher level of industry–government collaboration, and international coordination are critical for fighting the dark side of the metaverse.

## Data Availability

We do not analyse or generate any datasets, because our work proceeds within a theoretical approach.

## Footnotes

1 In this article, the author expresses his opinion and does not represent any organization that the author is associated with.
